# Paclitaxel targets FOXM1 to regulate KIF20A in mitotic catastrophe and breast cancer paclitaxel resistance

**DOI:** 10.1038/onc.2015.152

**Published:** 2015-05-11

**Authors:** P Khongkow, A R Gomes, C Gong, E P S Man, J W-H Tsang, F Zhao, L J Monteiro, R C Coombes, R H Medema, U S Khoo, E W-F Lam

**Affiliations:** 1 Department of Surgery and Cancer, Imperial College London, Hammersmith Hospital Campus, London, UK; 2 Department of Pathology, Li Ka Shing Faculty of Medicine, The University of Hong Kong, Hong Kong SAR, China; 3 Department of Clinical Oncology, Li Ka Shing Faculty of Medicine, The University of Hong Kong, Hong Kong SAR, China; 4 Division of Cell Biology, The Netherlands Cancer Institute, Amsterdam, The Netherlands

## Abstract

FOXM1 has been implicated in taxane resistance, but the molecular mechanism involved remains elusive. In here, we show that FOXM1 depletion can sensitize breast cancer cells and mouse embryonic fibroblasts into entering paclitaxel-induced senescence, with the loss of clonogenic ability, and the induction of senescence-associated β-galactosidase activity and flat cell morphology. We also demonstrate that FOXM1 regulates the expression of the microtubulin-associated kinesin KIF20A at the transcriptional level directly through a Forkhead response element (FHRE) in its promoter. Similar to FOXM1, KIF20A expression is downregulated by paclitaxel in the sensitive MCF-7 breast cancer cells and deregulated in the paclitaxel-resistant MCF-7Tax^R^ cells. KIF20A depletion also renders MCF-7 and MCF-7Tax^R^ cells more sensitive to paclitaxel-induced cellular senescence. Crucially, resembling paclitaxel treatment, silencing of FOXM1 and KIF20A similarly promotes abnormal mitotic spindle morphology and chromosome alignment, which have been shown to induce mitotic catastrophe-dependent senescence. The physiological relevance of the regulation of KIF20A by FOXM1 is further highlighted by the strong and significant correlations between FOXM1 and KIF20A expression in breast cancer patient samples. Statistical analysis reveals that both FOXM1 and KIF20A protein and mRNA expression significantly associates with poor survival, consistent with a role of FOXM1 and KIF20A in paclitaxel action and resistance. Collectively, our findings suggest that paclitaxel targets the FOXM1-KIF20A axis to drive abnormal mitotic spindle formation and mitotic catastrophe and that deregulated FOXM1 and KIF20A expression may confer paclitaxel resistance. These findings provide insights into the underlying mechanisms of paclitaxel resistance and have implications for the development of predictive biomarkers and novel chemotherapeutic strategies for paclitaxel resistance.

## Introduction

Breast cancer is the most common malignancy in women and a leading cause of mortality worldwide. Paclitaxel (also known as Taxol), together with docetaxel (Taxotere), belongs to the class of chemotherapeutic drugs called taxanes. They are commonly used as single agents or in combination with anthracyclines or radiotherapy for the treatment of breast cancers, in particular those not suitable for endocrine therapies as well as metastatic diseases.^1, 2, 3^ The primary mechanism of action of the taxanes is the disruption of microtubule (MT) dynamics through the stabilization of GDP-bound tubulin in the MT, thereby interrupting the process of cell division at mitosis. However, the efficiency of taxanes is often hampered by their toxic side effects, their poor solubility and the development of drug resistance in patients.^4, 5^ In addition, despite being one of the most widely used chemotherapeutics for solid tumours, the exact mechanisms and the factors that govern their anticancer functions are not completely understood.^6^

Cellular senescence is a tumour-suppressive phenomenon that limits unrestricted cell proliferation and in doing so, prevents cancer initiation and progression.^7^ Cells can be triggered to enter premature senescence by stress signals, including irradiation, persistent DNA damage response, oncogene activation, telomere erosion, oxidative stress, toxins and stem cell reprogramming.^7^ Mitotic catastrophe is a tumour-suppressive mechanism triggered during or after defective mitosis, culminating in senescence or cell death distinct from apoptosis.^8^ Conversely, defective mitotic catastrophe when coupled with mitotic slippage can promote genetic instability and tumourigenesis.^9^

FOXM1 is a member of the Forkhead box (FOX) family of transcription factors that share a characteristic winged-helix DNA-binding domain.^10^ It plays a central role in a variety of biological processes, including cell cycle progression, angiogenesis, metastasis, apoptosis, tissue regeneration and drug resistance. Additionally, FOXM1 is widely expressed in actively proliferating tissues and plays a key role in oncogenesis. Recent evidence also suggests FOXM1 can protect cells from genotoxic agent-induced senescence by enhancing DNA repair.^11, 12^ Consistently, FOXM1 is overexpressed in genotoxic agent-resistant cancer cells.^11, 13^ FOXM1 has been implicated in paclitaxel resistance but the exact mechanism by which FOXM1 modulates the anticancer effects of paclitaxel remains undefined.

Kinesins (also known as KIFs) are a superfamily of molecular motors engaged in key cellular functions including, mitosis, migration and intracellular transport, through their interaction with MTs.^14, 15, 16^ Kinesins are also believed to play a central role in mitosis during cell division through modulating MT dynamics.^17^ In here, we study the involvement of FOXM1 in paclitaxel drug action and resistance, and find that FOXM1 regulates KIF20A expression to modulate mitotic catastrophe, which has a role in paclitaxel-mediated cell death and senescence.

## Results

### Deletion of FOXM1 inhibits cell viability and induces cellular senescence in response to paclitaxel treatment

**Figure 1:**
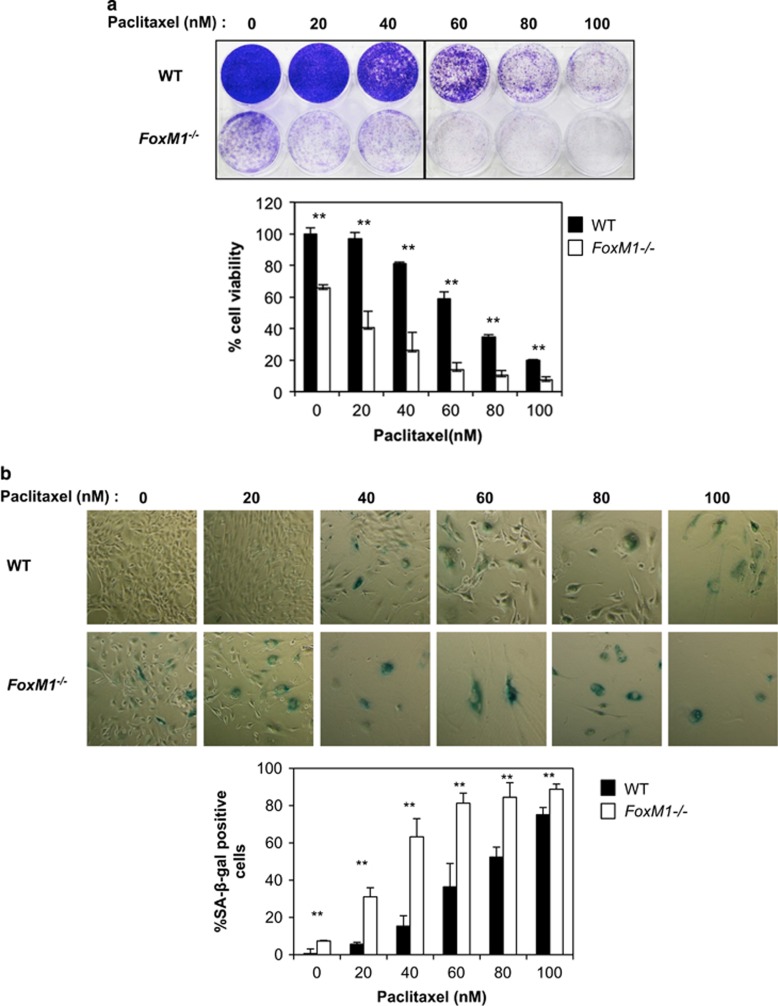
FOXM1 deletion inhibits cell proliferation and induces cellular senescence in response to paclitaxel treatment in MEFs. (**a**) Clonogenic assay was performed to assess the colony formation efficiency of *FoxM1*^*−/−*^ and WT MEFs. Two thousand cells were seeded in six-well plates, treated with 0, 20, 40, 60, 80 and 100 nM of paclitaxel and grown for 15 days. The cells were then stained with crystal violet. Representative results are shown. The bar graph represents an average of three independent experiments±s.d. (*n*=3). Statistical significance was determined by Student's *t*-test, two-sided (*t*-test: *FoxM1*^*−/−*^ versus WT MEFs. Significant **P⩽0.01). (**b**) SA-β-gal staining of *FoxM1*^*−/−*^ and WT MEFs. The MEFs were treated with 0, 20, 40, 60, 80 and 100 nM of paclitaxel and then stained for SA-β-gal activity 5 days after treatment. The bar graph represents an average of three independent experiments±s.d. (*n*=3). Statistical significance was determined by Student's *t*-test, two-sided (*t*-test: *FoxM1*^*−/−*^ versus WT MEFs. Significant **P⩽0.01).

Our previous research implicated a role of FOXM1 in modulating taxane sensitivity.^18^ To establish a role of FOXM1 in the response to paclitaxel, we evaluated the long-term cell viability of early passage wild-type (WT) and *FoxM1*^*−/−*^ mouse embryonic fibroblasts (MEFs) by clonogenic assay upon treatment with a range of concentrations of paclitaxel. The results showed that *FoxM1*^*−/−*^ MEFs were significantly more sensitive to paclitaxel compared with WT MEF cells (Figure 1a). To determine whether this loss of long-term viability is due to cellular senescence, the WT and *FoxM1*^*−/−*^ MEFs were subjected to senescence-associated (SA) β-galactosidase (β-gal) staining. In agreement, the results indicated that FOXM1 deletion in MEFs significantly enhanced senescence upon paclitaxel treatment, as revealed by their increased β-gal staining and flat cell morphology (Figure 1b).

### KIF20A and FOXM1 mRNA and protein display similar kinetics in both MCF-7 and paclitaxel-resistant MCF-7 Tax^R^ cells following paclitaxel treatment

**Figure 2:**
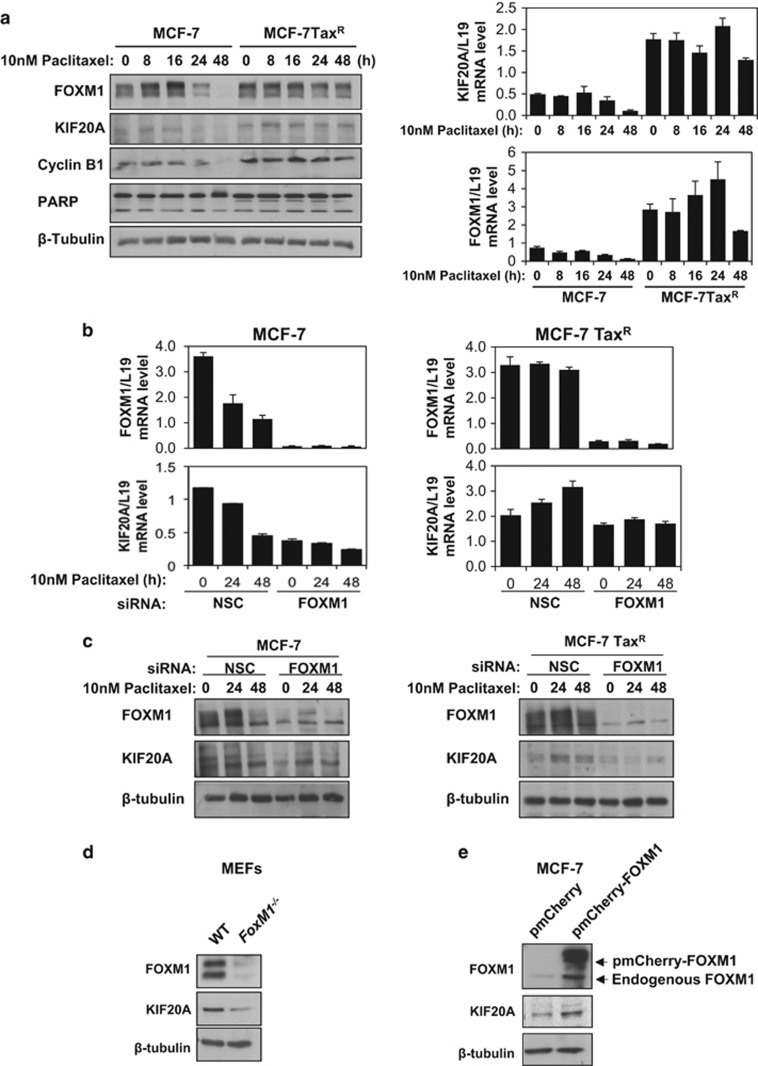
Paclitaxel-resistant MCF-7 cells exhibit upregulated expression levels of FOXM1 and KIF20A. (**a**) Protein expression levels of FOXM1, KIF20A, cyclin B1 and PARP in MCF-7 and MCF-7 Tax^R^ cell lines were examined by western blotting after paclitaxel treatment at different time points indicated (left panel). Notably, there was no cleavage of PARP. qRT-PCR analysis determining the relative mRNA expression levels of FOXM1 and KIF20A in MCF-7 and MCF-7 Tax^R^ cell lines after paclitaxel treatment. The bar graph represents an average of three independent experiments±s.d. (*n*=3). (**b**) MCF-7 and MCF-7 Tax^R^ cells transfected with Smart pool siRNA against FOXM1 or with non-silencing controls (NSC) were treated with 10 nM paclitaxel and harvested for FOXM1 and KIF20A expression analysis. FOXM1 and KIF20A mRNA levels were determined by qRT-PCR. Value is mean ±s.d. (*n*=3). (**c**) The expression levels of FOXM1, KIF20A and β-tubulin were analysed by western blot analysis. (**d**) The expression levels of FOXM1, KIF20A and β-tubulin were analysed by western blot analysis in *FoxM1*^*−/−*^ and WT MEFs. (**e**) The expression levels of FOXM1, KIF20A and β-tubulin were also analysed by western blot analysis in MCF-7 cells transfected with control vector and the pmCherry-FOXM1 expression vector.

The kinesin KIF20A has been shown to be a potential downstream FOXM1 target required for normal spindle formation and chromosome segregation.^19^ To explore a possible role of FOXM1 in paclitaxel resistance and the mechanism of action involved, we investigated the expression levels of FOXM1 and its putative target KIF20A in the breast carcinoma MCF-7 cells as well as the paclitaxel-resistant MCF-7 Tax^R^ cells in response to paclitaxel treatment. Western blot analysis showed that FOXM1 expression was downregulated in the sensitive MCF-7 cells in response to moderate levels of paclitaxel (10 nM), while the expression levels of FOXM1 were maintained at high levels in the MCF-7 Tax^R^ cells upon paclitaxel treatment. Intriguingly, the expression of KIF20A followed similar kinetics as FOXM1 upon paclitaxel treatment in both cell lines, indicating a potential role for FOXM1 in modulating paclitaxel sensitivity through KIF20A (Figure 2a; left panel, Supplementary Figure S1). Consistently, RT-qPCR analysis revealed that both FOXM1 and KIF20A mRNA levels were increased by two to threefold in MCF-7 Tax^R^ cells compared with the parental MCF-7 cells, which exhibited a reduction in KIF20A transcript levels following paclitaxel treatment (Figure 2a; right panel). Together these results suggest that FOXM1 regulates KIF20A to modulate paclitaxel sensitivity in breast cancer.

### Downregulation of FOXM1 decreases the levels of KIF20A, a kinesin involved in mitotic progression

To determine whether KIF20A is a downstream target of FOXM1, we profiled the expression of KIF20A by RT-qPCR and western blot analysis after silencing FOXM1 using short interfering RNA (siRNA) in paclitaxel-treated MCF-7 and MCF-7 Tax^R^ cells. The results showed that depletion of FOXM1 culminated in the downregulation of KIF20A at both the mRNA and protein levels (Figures 2b and c, Supplementary Figure S2), suggesting FOXM1 regulates KIF20A expression. Notably, both FOXM1 and KIF20A are induced at the protein levels after paclitaxel treatment, which is likely owing to the fact both proteins are upregulated at the post-transcriptional levels in mitotsis. Consistently, both FOXM1 and KIF20A have been shown to be upregulated by mitotic inhibitors at the post-translational levels.^18, 20, 21^ In agreement, KIF20A levels were also detected at lower levels in *FoxM1*^*−/−*^ MEFs compared with WT MEFs (Figure 2d) as well as in MDA-MB-231 breast cancer cells after FOXM1 depletion (Supplementary Figure S3). Conversely, ectopic overexpression of FOXM1 in MCF-7 cells augmented the expression of KIF20A (Figure 2e).

### FOXM1 enhances KIF20A promoter activity in MCF-7 cells

**Figure 3:**
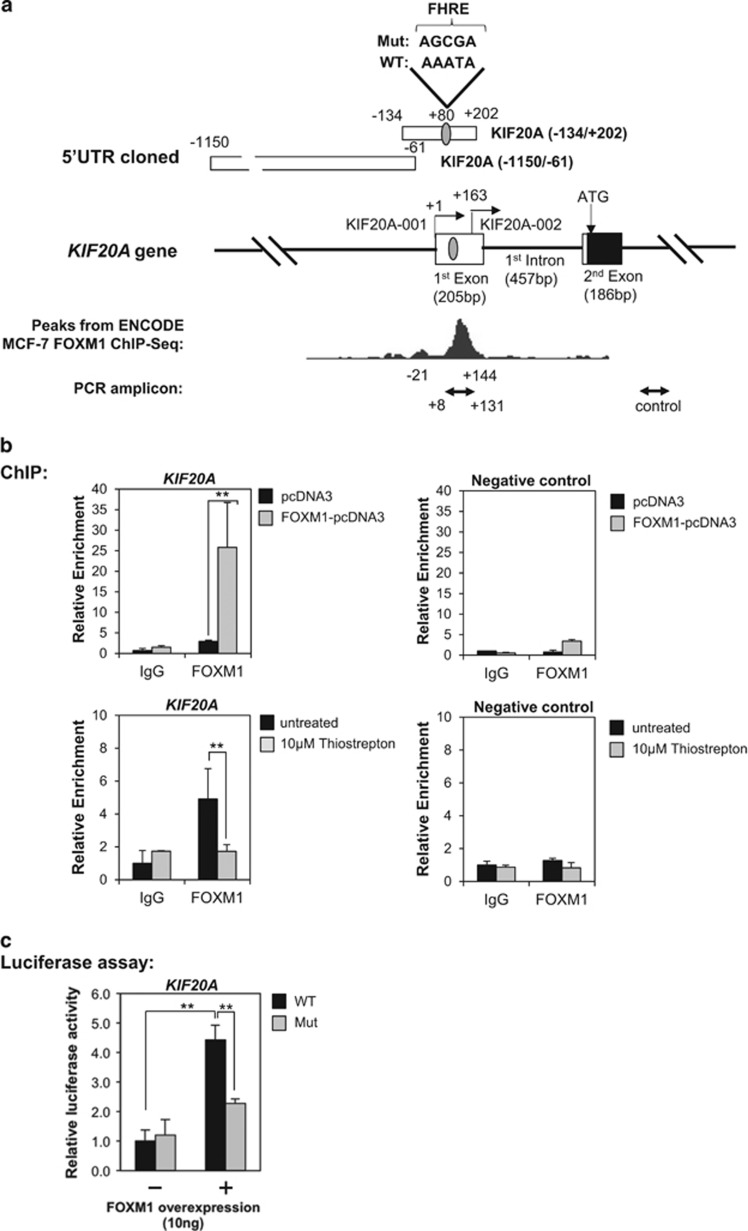
FOXM1 binds to the *KIF20A* promoter region in MCF-7 breast cancer cell line. (**a**) Schematic representation of the 5' region of the human *KIF20A* gene depicting the FHRE, the putative promoter regions cloned, the WT and the mutant (mut) promoter sequences, the ENCODE ChIP-seq data and the ChIP-qPCR amplicon. (**b**) The binding of FOXM1 to the human *KIF20A* promoter was examined by ChIP analysis in MCF-7 cells transfected with control vector or a FOXM1 expression vector (upper panel). The occupancy of the FHRE by FOXM1 was also examined in MCF-7 with and without 10μM thiostrepton treatment. (**c**) MCF-7 cells were transiently co-transfected with WT or mut *KIF20A* reporter plasmids and Renilla luciferase plasmid along with or without FOXM1 expression plasmids (10 μg). Twenty-four hours after transfection, cells were lysed, and the luciferase activity examined. Firefly luminescence signal was normalized with the Renilla luminescence signal. Value is mean±s.d. (*n*=3). Statistical significance was determined by Student's *t*-test, two-sided (very significant **P⩽0.01).

To determine whether FOXM1 is a direct upstream transcriptional activator of KIF20A, we sought to clone the *KIF20A* promoter. We initially cloned a 1.1 kbp region (−1150/−61) upstream of the most 5'-transcription start site (designated +1 bp; Esembl KIF20A-001 transcript) (Figure 3a). However, this 5'-UTR region of KIF20A failed to demonstrate significant responsiveness to *trans*activation by FOXM1 in promoter/luciferase reporter assays (Supplementary Figure S4). We thus next analysed the MCF-7 ChIP-Seq data (hg19: GSM1010769) from the Encyclopedia of DNA Elements (ENCODE) project^22^ and identified strong FOXM1 occupancy at a region (−21/+144) mapped downstream of the most 5'-transcription start site but upstream of a second transcription start site (designated+163; Esembl KIF20A-002 transcript) (Figure 3a). Sequence analysis also identified a putative forkhead responsive element (FHRE) (+80 bp) within this region (Figure 3a). To determine whether FOXM1 directly binds to the *KIF20A* promoter region, we performed chromatin immunoprecipitation (ChIP) analysis in MCF-7 cells using specific primers (+8/+133) to amplify the region containing the putative FHRE (Figure 3b) and analysed FOXM1-binding using RT-qPCR. As shown in Figure 3b (top left panel), the ChIP analysis showed that overexpression of FOXM1 enhances the binding of FOXM1 to the *KIF20A* promoter region. Conversely, the inhibition of FOXM1 binding by thiostrepton significantly decreased the FOXM1 occupancy (Figure 3b, bottom left panel). To test whether FOXM1 can transactivate this *KIF20A* region through the FHRE, MCF-7 cells were transiently co-transfected with a FOXM1 expression construct and a luciferase reporter gene under the control of either a WT or a mutant (mut) *KIF20A* (0.3Kbp; −134/+202 bp) sequence (Figure 3a). The results showed that the WT *KIF20A* promoter activity was significantly augmented by FOXM1, whereas the mutant (mut) *KIF20A* promoter was not *trans*activated by FOXM1 (Figure 3c), suggesting FOXM1 can activate KIF20A transcription through this FHRE. Collectively, these results suggest that FOXM1 is able to bind and transactivate the *KIF20A* gene through the FHRE located at position −80 bp, providing strong indication that FOXM1 is a direct upstream transcriptional regulator of *KIF20A*.

### Low doses of paclitaxel cause aberrant mitosis in MCF-7 cells

**Figure 4:**
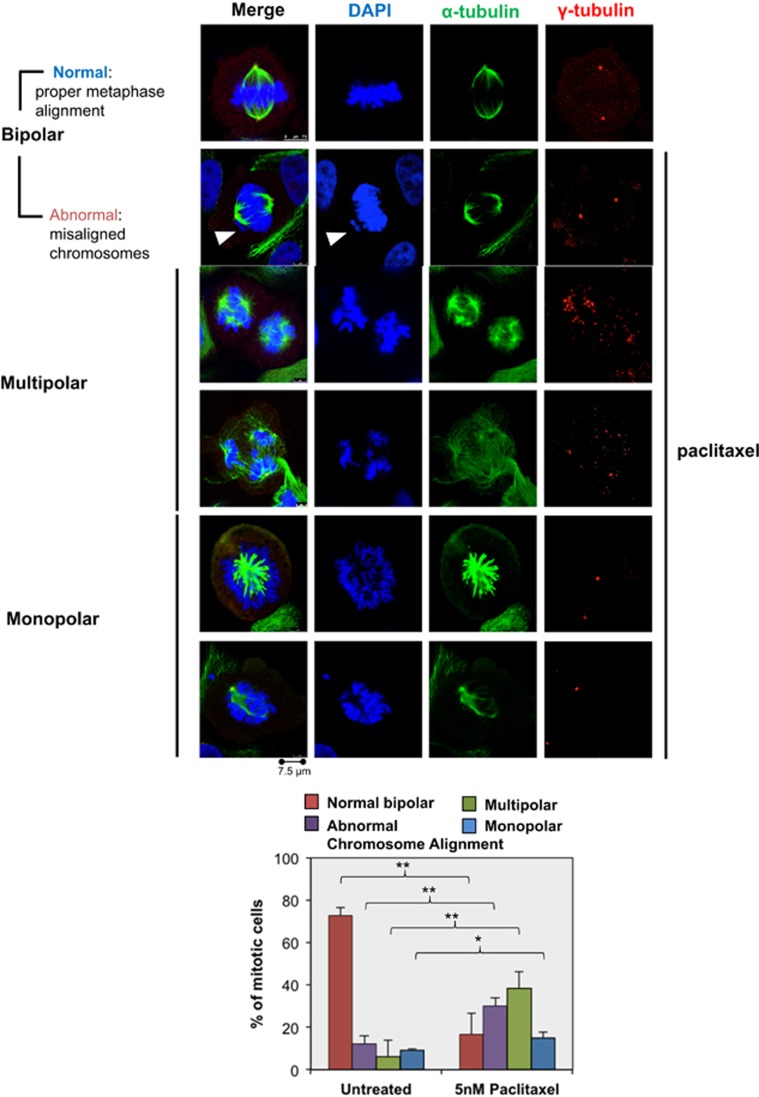
Low-dose paclitaxel induces mitotic catastrophe. MCF-7 cells cultured on chamber slides were treated with or without 5 nM paclitaxel for 24 h. Cells were then fixed, permeabilized, and immunostained with antibody against α-tubulin (Green) and γ-tubulin (Red). Nuclei were counterstained with 4',6-diamidino-2-phenylindole (Blue). Mitotic cells were visualized with Leica TCS SP5 ( × 63 magnification). For each condition, images of at least 50 mitotic cells were captured. Representative confocal images with and without 5 nM paclitaxel treatment are shown (upper panel). Arrows indicate misaligned chromosomes. The number of mitotic cells classified into either normal bipolar, abnormal misaligned chromosome, monopolar or multipolar spindles was quantified. Results represent the mean of three independent sets of experiments±s.d. (lower panel). Statistical significance was determined by Student's *t*-test, two-sided (significant, *P⩽0.05; very significant **P⩽0.01).

To determine the cellular consequences of paclitaxel treatments, MCF-7 cells were treated with a low dose of paclitaxel (5 nM) and mitotic spindle formation was examined using α-tubulin antibodies to stain MTs, γ-tubulin antibodies to identify the centrosomes and 4',6-diamidino-2-phenylindole to stain DNA. Figure 4 (top panel) illustrates a typical untreated cell in mitosis (metaphase), with a normal bipolar spindle. Figure 4 also (lower panels) reveals various mitotic abnormalities, including abnormal chromosome segregation, monopolar and multipolar spindles, found in paclitaxel-treated MCF-7 cells. Quantitative analysis of mitotic cells stained with α-tubulin and γ-tubulin antibodies (Figure 4) indicates that over 80% of paclitaxel-treated MCF-7 cells exhibit abnormal mitotic spindles, with significant increases in cells with abnormal monopolar and multipolar spindles as well as chromosome misalignment.

### Depletion of FOXM1 or KIF20A causes abnormal mitotic spindle formation and chromosome alignment defects in MCF-7 cells

**Figure 5:**
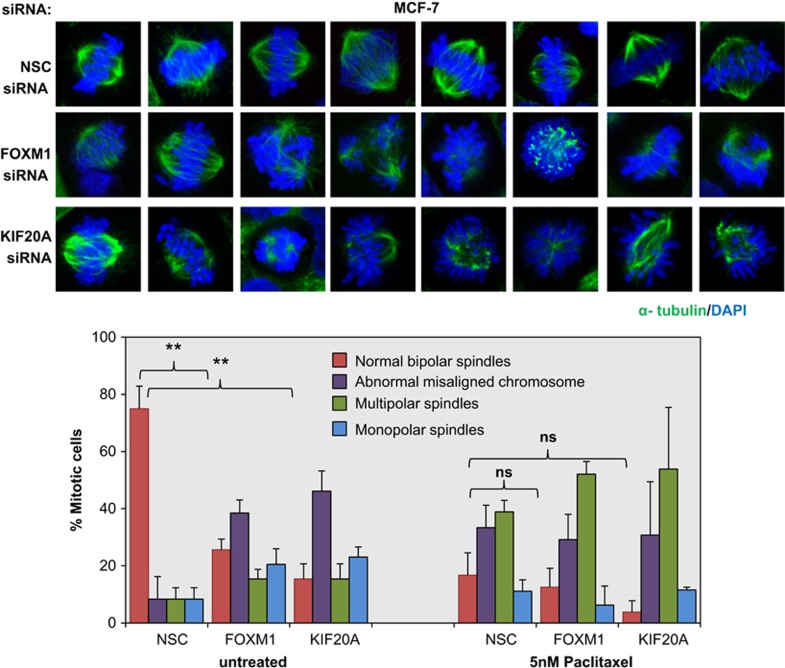
Characterization of mitotic spindle defects in MCF-7 cells following FOXM1 or KIF20A depletion. MCF-7 cells were transfected with non-silencing controls (NSC), FOXM1 or KIF20A siRNA. Twenty-four hours after transfection, cells cultured on chamber slides were either untreated or treated with 5 nM paclitaxel for 24 h. Cells were then fixed, permeabilized and immunostained with antibody against α-tubulin (Green) and γ-tubulin (Red). Nuclei were counterstained with 4',6-diamidino-2-phenylindole (Blue). Mitotic cells were visualized with Leica TCS SP5 ( × 63 magnification). For each condition, images of at least 50 mitotic cells were captured. Representative confocal images are shown. The number of mitotic cells classified into either normal bipolar, bipolar with chromosome misalignment, monopolar or multipolar spindles was quantified. Results represent average of three independent experiments±s.d. Statistical significance was determined by Student's *t*-test, two-sided (not significant, ns; very significant **P⩽0.01).

To study the mitotic defects induced by loss of FOXM1 and KIF20A, the subcellular distribution of α-tubulin and chromosomes was examined in metaphase of MCF-7 cells after knockdown of FOXM1 or KIF20A (Figure 5; Supplementary Figure S5). In the majority of the control siRNA-transfected cells, condensed chromosomes were aligned properly at the metaphase plate with bipolar spindles. By contrast, in both the FOXM1 and KIF20A-depleted cells, there was a significant increase in monopolar and multipolar mitotic spindles as well as bipolar spindles with misaligned chromosomes. The frequency of abnormal mitotic spindles in MCF-7 cells was increased approximately threefold compared with the control in FOXM1 knockdown and about fourfold in KIF20A knockdown cells. These failures to establish normal mitotic spindles in metaphase induced by KIF20A and FOXM1 depletion also caused a significant increase lagging chromosomes in anaphase (Supplementary Figure S6) and ultimately, the accumulation of large multinucleated and micronucleated cells, indicative of mitotic catastrophe (Supplementary Figure S7).^9, 19, 23^ These results indicated that FOXM1 and KIF20A are essential for the formation of normal mitotic spindles, and defects of which lead to abnormal chromosome segregation and mitotic progression. Intriguingly, in the paclitaxel-treated MCF-7 cells, the increase in abnormal spindle formation after FOXM1 or KIF20A silencing was no longer apparent. Consistent with previous results, together these findings suggest that FOXM1 and KIF20A modulate the cytostatic and cytotoxic function of paclitaxel through regulation of mitotic spindle formation.

### Depletion of KIF20A or FOXM1 inhibits cell growth and induces senescence in MCF-7 cells

**Figure 6:**
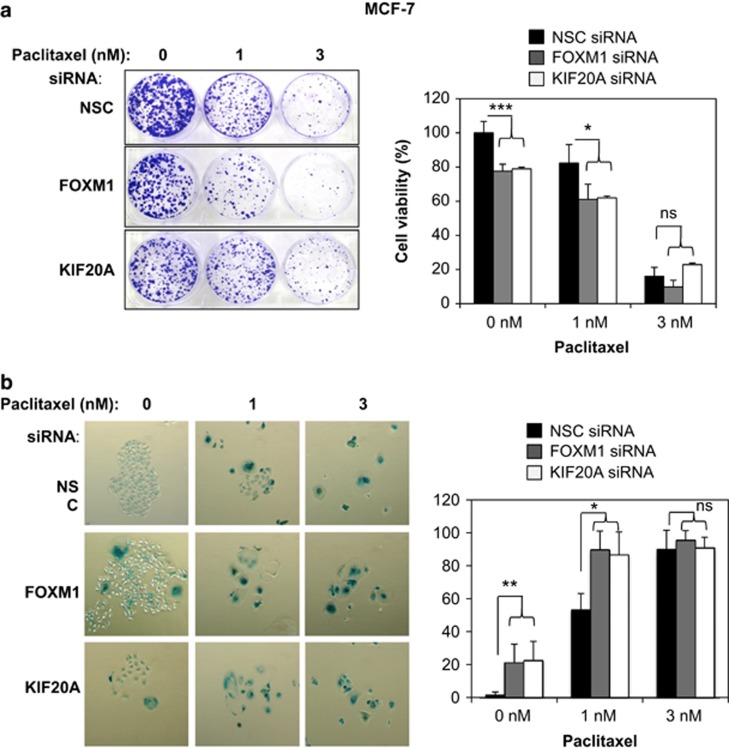
Depletion of KIF20A or FOXM1 inhibits cell proliferation and induces senescence in MCF-7 cells. (**a**) MCF-7 cells were transfected with either non-silencing control (NSC) siRNA, siRNA targeting FOXM1 or KIF20A. Twenty-four hours after transfection, 2000 cells were seeded in six-well plates, treated with 0, 1 or 3 nM of paclitaxel, grown for 15 days, and then stained with crystal violet (left panel). The result (right panel) represents an average of three independent experiments±s.d. Statistical significance was determined by Student's *t*-test, two-sided (*P⩽0.05, **P⩽0.01, ***P⩽0.005; n.s., non-significant). In parallel, (**b**) MCF-7 transfected with NSC, FOXM1 or KIF20A siRNA were seeded in six-well plates, treated with 0, 1 or 3 nM of paclitaxel. Five days after treatment, cells were stained for SAβ-gal activity. The graph shows the percentage of SAβ-gal-positive cells as measured from five different fields from three independent experiments. Bars represent mean±s.d. Statistical significance was determined by Student's *t*-test, two-sided (*P⩽0.05, **P⩽0.01, ***P⩽0.005, significant; n.s., non-significant).

**Figure 7:**
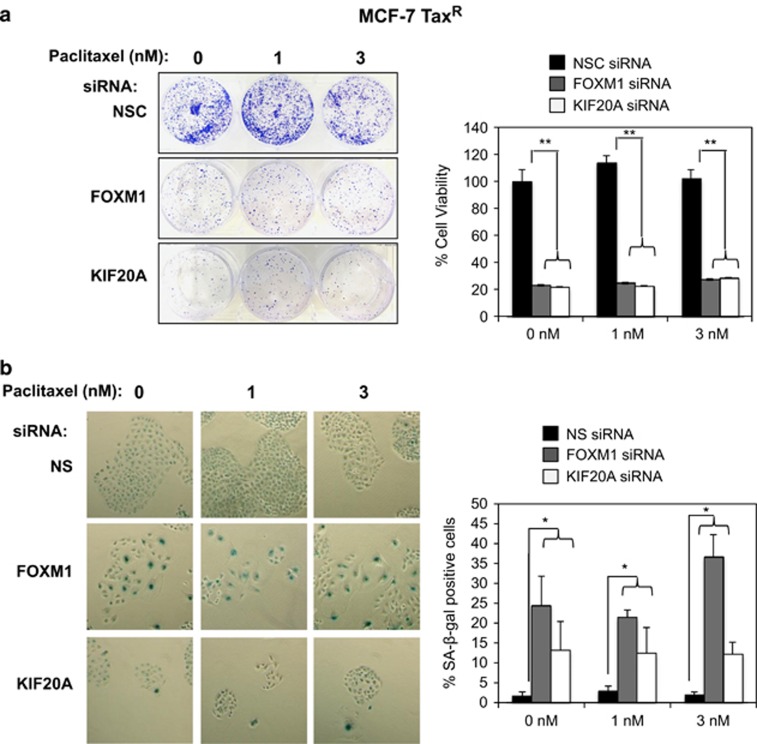
Knockdown of FOXM1 or KIF20A suppresses cell proliferation and induces senescence in MCF-7 Tax^R^ cells. (**a**) MCF-7 Tax^R^ were transfected with either non-silencing control (NSC) siRNA, siRNA targeting FOXM1 or KIF20A. Twenty-four hours after transfection, 2000 cells were seeded in six-well plates, treated with 0, 1 or 3 nM of paclitaxel, grown for 15 days and then stained with crystal violet (left panel). The result (right panel) represents an average of three independent experiments±s.d. Statistical significance was determined by Student's *t*-test, two-sided (*P⩽0.05, **P⩽0.01, significant; n.s., non-significant). In parallel, (**b**) MCF-7 Tax^R^ transfected with NSC, FOXM1 or KIF20A siRNA were seeded in six-well plates, treated with 0, 1 or 3 nM of paclitaxel. Five days after treatment, cells were stained for SAβ-gal activity. The graph shows the percentage of SAβ-gal-positive cells as measured from five different fields from three independent experiments. Bars represent average±s.d. Statistical significance was determined by Student's *t*-test, two-sided (*P⩽0.05, **P⩽0.01, ***P⩽0.005, significant; n.s., non-significant).

We next tested the effects of targeting FOXM1 and KIF20A in MCF-7 and the paclitaxel-resistant MCF-7 Tax^R^ breast cancer cell lines. To this end, cells were transfected with siRNA pools targeting FOXM1 or KIF20A and their proliferation rates evaluated by clonogenic assays. The results showed that FOXM1-knockdown sensitized MCF-7 cells to long-term proliferative arrest at relatively low paclitaxel doses (for example, 1 and 3 nM) (Figure 6a, Supplementary Figures S8 and S9). This notion is supported by the analogous results from FoxM1-null fibroblasts (Figure 1) and the observation that ectopic FOXM1 expression conferred paclitaxel resistance to MCF-7 cells (Supplementary Figure S10). Similar to FOXM1 depletion, knockdown of KIF20A also sensitized MCF-7 cells to paclitaxel at very low concentrations of the drug (Figure 6a). In agreement with the clonogenic assay results, FOXM1 or KIF20A knockdown sensitized MCF-7 cells to paclitaxel-induced senescence, as revealed by the accumulation of cells displaying the SA β-gal activity and flat cell morphology (Figure 6b). Interestingly, knockdown of FOXM1 or KIF20A alone almost completely abolished the colony-forming capacity of MCF-7 Tax^R^ cells irrespective of the dosage of paclitaxel used, suggesting that MCF-7 Tax^R^ cells are dependent on high expression levels of FOXM1 and KIF20A for long-term clonal survival (Figure 7a, Supplementary Figures S8 and S11). Depletion of FOXM1 or KIF20A in MCF-7 Tax^R^ cells significantly induced the SA β-gal activity and morphology independent of the paclitaxel concentration, suggesting MCF-7 Tax^R^ cells have become dependent on FOXM1 and KIF20A expression to override the senescence programme (Figure 7b).

### Correlation between KIF20A and FOXM1 expression in breast cancer samples

**Figure 8:**
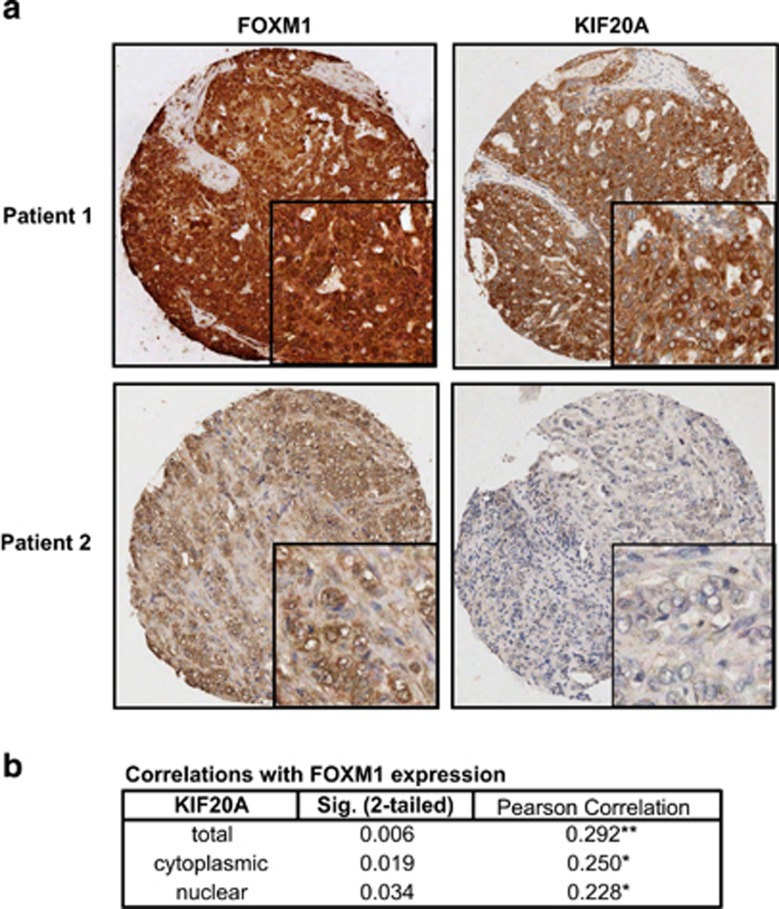
(**a**) FOXM1 and KIF20A expression was assessed by immunohistochemistry using tissue-microarray constructed from 116 breast cancer patient samples. KIF20A was expressed in both cytoplasm and nucleus. Representative staining images of one patient with high FOXM1 and KIF20A expression and one with low expression are shown. Positive correlation between FOXM1 and KIF20A was observed. (**b**) KIF20A staining were detected in both nuclear and cytoplasmic compartments and were correlated with FOXM1 staining. Statistical analysis revealed that all three KIF20A scores (total, cytoplasmic and nuclear) were significantly correlated with FOXM1 expression. (*P*=0.006, *P*=0.019 and *P*=0.034, respectively). *P*⩽0.05, significant; *P*⩽0.01, very significant; *P*⩽0.005, very very significant.

**Figure 9:**
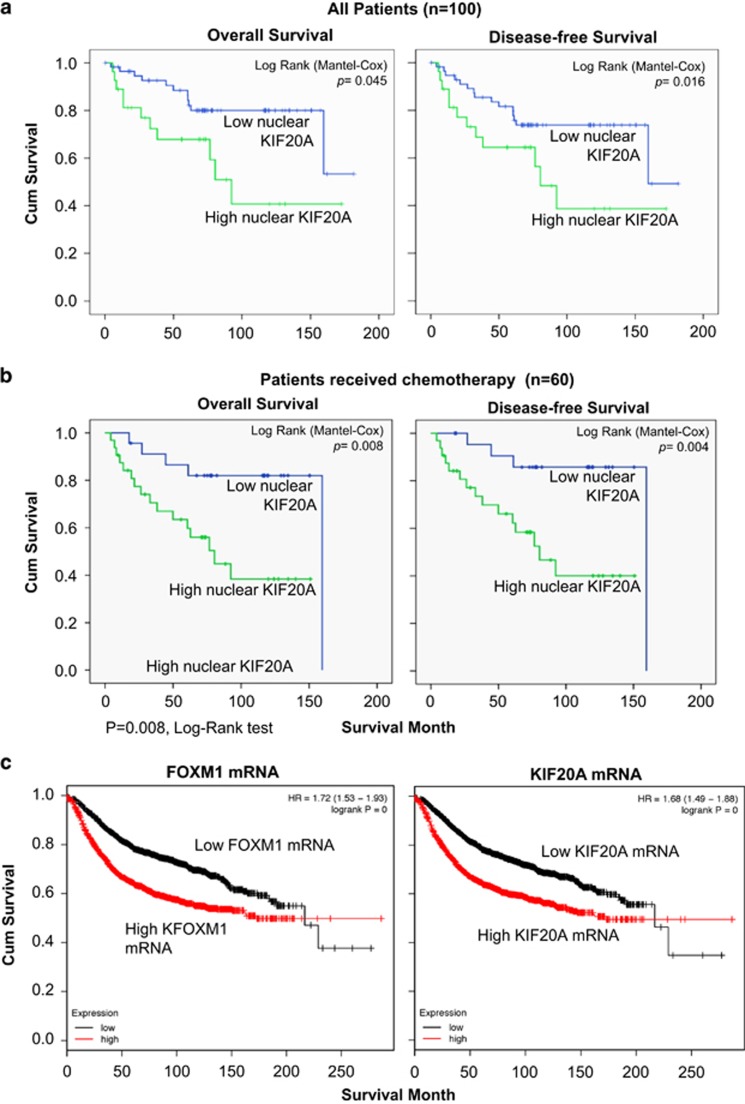
KIF20A overexpression significantly associated with poorer survival in breast cancer patients. (**a**) Kaplan–Meier survival analysis (SPSS) of all patients showed that nuclear KIF20A overexpression significantly associated with poorer survival (*n*=100). (**b**) Kaplan–Meier survival analysis of patients received chemotherapy showed that nuclear KIF20A overexpression significantly associated with poorer survival (*n*=60). (**c**) Kaplan–Meier survival analysis of patients FOXM1 and KIF20A mRNA overexpression significantly associated with poorer survival (*n*=3455). *P*⩽0.05, significant; *P*⩽0.01, very significant; *P*⩽0.005, very very significant.

To establish further the physiological significance and clinical relevance of the regulation of KIF20A by FOXM1 in breast cancer, FOXM1 and KIF20A expression was assessed by immunohistochemistry in 116 breast cancer patient samples (Figure 8a). Immunohistochemical analysis results revealed FOXM1 expression significantly correlated with KIF20A expression (Pearson coefficient *r*=0.292, *P*=0.006 for total KIF20A; *r*=0.250, *P*=0.019 for cytoplasmic KIF20A; *r*=0.228, *P*=0.034 for nuclear KIF20A) (Figure 8b). This further strengthened our finding in the cell lines that FOXM1 directly regulates KIF20A transcription. Moreover, survival analysis showed that nuclear KIF20A overexpression significantly associated with poorer survival (log-rank test, *P*=0.045 for overall survival and *P*=0.016 for disease-specific survival, respectively) (Figure 9a; Supplementary Figure S12). On multivariate analysis, KIF20A nuclear staining remained associated with poor survival after correcting for tumour stage and lymph-node involvement (*P*=0.047, relative risk=2.47 for overall survival and *P*=0.037, relative risk=2.767 for disease-specific survival, respectively) (Supplementary Figure S12), supporting that KIF20A nuclear score is a prognostic marker independent of the clinicopathological parameters examined. In this cohort, 60% of patients received chemotherapy. For these patients, elevated nuclear KIF20A was significantly associated with poor survival (log-rank test, *P=*0.008 for overall survival and *P*=0.004 for disease-specific survival, respectively) (Figure 9b; Supplementary Figure S13) and is an even stronger risk marker (*P*=0.013, relative risk=4.008 for overall survival and *P*=0.01, relative risk=5.089 for disease-specific survival, respectively) (Supplementary Figure S13), suggesting that similar to FOXM1,^11^ KIF20A expression is associated with chemotherapeutic drug resistance. The fact that only nuclear KIF20A is a reliable prognostic marker suggests further post-translational mechanisms modulate its nuclear oncogenic function. In agreement, further analysis of KIF20A and FOXM1 transcript expression in a previously published cohort (3455 breast cancer patients)^24^ revealed that both high FOXM1 and KIF20A mRNA expression levels are very significantly associated with poor survival (*P*<0.00001 and *P*<0.00001, respectively, for overall survival, Kaplan–Meier analysis) (Figure 9c). The significance of both FOXM1 and KIF20A in survival analyses provides further evidence for the involvement of both genes in breast cancer progression and drug response.

## Discussion

Mitotic spindles are responsible for the proper distribution of newly duplicated chromosomes to the two nascent daughter cells during mitosis.^25^ The processes for spindle assembly and function as well as sister chromatid segregation are modulated by MT polarity and dynamics. MT stabilizers, including paclitaxel, suppress MT dynamics and activate the mitotic checkpoint, causing cell proliferation arrest and/or cell death. We found that FOXM1 is overexpressed in paclitaxel-resistant MCF-7 Tax^R^ breast cancer cell lines when compared with the parental sensitive MCF-7 cells. FOXM1 expression is downregulated in response to paclitaxel in MCF-7 cells, but remains persistently high in the resistant cells following paclitaxel treatment. These data suggest the possibility that FOXM1 is a target of paclitaxel and that it has a role in mediating paclitaxel action and resistance. Consistent with this idea, we have shown previously that paclitaxel mediates its cytotoxic functions through FOXO3a,^26, 27^ which is an upstream negative regulator of FOXM1 expression and activity.^10, 28, 29^ Interestingly, our data also reveal that in response to paclitaxel, breast cancer cell lines undergo mitotic catastrophe, followed by cellular senescence and/or non-apoptotic cell death. Crucially, similar to paclitaxel treatment, FOXM1 depletion also induces mitotic catastrophe, culminating in non-apoptotic cell death and senescence. Consistent with this, previous studies also shows that loss of FOXM1 can induce chromosome misalignment, centrosome amplification and mitotic catastrophe.^19, 30^

The assembly of mitotic spindle and the subsequent chromosome segregation are facilitated by MT-associated kinesins.^17, 31^ These enzymes convert chemical energy of ATP hydrolysis into mechanical force production to mobilize MTs during mitosis. KIF20A, also known as MKlP2 (mitotic kinesin-like protein 2) or Rab6 kinesin, is a MT-associated motor protein of the kinesin-6 subfamily that regulates mitosis and cytokinesis.^32, 33^ We found that *KIF20A* is transcriptionally activated by FOXM1. In agreement, depletion of FOXM1 by siRNA downregulates KIF20A expression and paclitaxel downregulates FOXM1 and therefore, KIF20A expression in MCF-7 breast cancer cell lines. Our data also show that FOXM1 regulates KIF20A expression at the gene promoter level via a FHRE. Interestingly, our gene promoter and ChIP analyses reveal that this FHRE is located downstream of the most 5'-transcription start site, within the first non-coding exon, suggesting FOXM1 drives the transcription of *KIF20A* from this alternative promoter region in breast cancer cells. This finding is supported by a recent published global FOXM1 ChIP-sequence analysis in MCF-7 breast carcinoma cells (hg19: GSM1010769) from ENCODE project.^22^

Notably, the expression of KIF20A, like FOXM1, is downregulated in the drug-sensitive MCF-7 cells in response to paclitaxel treatment, suggesting further that both FOXM1 and KIF20A may mediate the paclitaxel action. Consistent with this idea, we found that the normal spindle structure and chromosome alignment were significantly disrupted after the depletion of KIF20A or FOXM1 using siRNA. However, the paclitaxel-induced spindle abnormalities and chromosome misalignment defects are not further enhanced by depletion of KIF20A or FOXM1, further confirming the idea that paclitaxel targets the FOXM1-KIF20A axis to induce mitotic catastrophe. Interestingly, our data also show an increase in mitotic catastrophe which can occur spontaneously in some FOXM1- or KIF20A-depleted cells, resembling paclitaxel treatment. This further suggests that paclitaxel mediates its function through targeting FOXM1 and KIF20A. The spindle assembly checkpoint monitors the correct attachment of MTs to the kinetochores of sister chromatids, and the detection of abnormal spindles by spindle assembly checkpoint will trigger mitotic catastrophe.^34^ We therefore speculate that the overexpression of FOXM1 and KIF20A observed in the paclitaxel-resistant cells counteracts the ability of paclitaxel to induce abnormal spindles through their downregulation.

Our immunohistochemical and statistical analysis of breast cancer patient samples shows that there is a significant and strong correlation between FOXM1 and KIF20A expression, further confirming the regulation of KIF20A by FOXM1 *in vivo*. Crucially, like FOXM1,^11^ the overexpression of nuclear KIF20A (Figure 9) is associated with poor prognosis in terms of overall and disease-specific survival. As over half of these patients studied have received chemotherapy in the forms of anthracyclins and taxanes,^11, 35^ the immunohistochemistry data also support the idea that FOXM1 regulates KIF20A to modulate paclitaxel resistance. In addition, these data also underscore the value of FOXM1 and KIF20A as biomarkers for the prediction of breast cancer chemotherapy sensitivity and patient survival. In summary, our data suggest that paclitaxel targets FOXM1 to downregulate kinesins such as KIF20A to interfere with the formation of the normal mitotic spindle, thus inducing senescence-related cell cycle arrest and cell death in these cells. The reason why depletion of KIF20A leads to spindle defects remains unclear. However, a recent study using a Xenopus egg cell free system shows that KIF20A is required for the transport of the chromosomal passenger complex, which in turn is implicated in the coordination of chromosome segregation.^33^ Collectively, these data advocate the idea that the FOXM1 may modulate paclitaxel sensitivity through regulating the expression levels of kinesins, such as KIF20A, involved in mitosis and cytokinesis.

Notably, while depletion of FOXM1 or KIF20A enhances the ability of paclitaxel to induce cellular senescence in MCF-7 cells, FOXM1 or KIF20A silencing can readily induce senescence in the resistant MCF-7 Tax^R^ cells. This implies that the resistant cells may have become over-reliant on FOXM1 and KIF20A for their long-term survival and renewal. As a consequence, the induction of abnormal spindle formation through FOXM1 and its downstream target KIF20A, may represent a novel strategy for overriding taxane resistance as well as for cancer treatment, as ensuing aberrant mitosis will culminate in senescence and/or cell death. Indeed, the thiazole antibiotics thiostrepton, which inhibits the transcriptional activity of FOXM1, has been shown to specifically target cancer cells and have lesser effects on non-cancerous cells.^36^ A reversible specific small molecule inhibitor of KIF20A called paprotrain [(Z)-2-(1H-indol-3-yl)-3-(pyridin-3-yl)acrylonitrile] has also been developed. In addition to paprotrain, a vaccine against peptides derived from KIF20A has also been used in a recent phase I immunotherapy clinical trial for advanced pancreatic cancer.^37^ In line with our findings, cells treated with paprotrain also accumulate at metaphase and anaphase and display an increased percentage of monopolar as well as multipolar spindles.^38, 39^ Interestingly, paprotrain has also been shown to inhibit tumour angiogenesis and development, independent of its mitotic function.^40^

In summary, we identify KIF20A as a direct transcriptional target of FOXM1, involved in paclitaxel action and resistance. We show that paclitaxel targets the FOXM1-KIF20A axis to induce mitotic catastrophe, and the deregulation of this axis may contribute to taxane resistance. Our data also suggest that FOXM1 and KIF20A can be useful predictive biomarkers, in addition to being therapeutic targets for cancer treatment and for tackling taxane resistance in cancer.

## Materials and methods

### Cell culture

The human breast carcinoma MCF-7 and MDA-MB-231 cell lines were originated from the American Type Culture Collection (Manassas, VA, USA) and were acquired from the Cell Culture Service, Cancer Research UK (London, UK), where it was tested and authenticated. The MCF-7 TaxR cell line was previously established in the lab by growing parental MCF-7 cells in stepwise-increasing paclitaxel concentrations until they acquired resistance to 100 μmol/l paclitaxel^35^ (Teva UK Limited, East Sussex, UK). The WT and *FoxM1*^*−/−*^ MEFs have previously been described.^30^ All cells were cultured in Dulbecco's modified Eagle's medium supplemented with 10% foetal calf serum, 2 mM glutamine, 100 U/ml penicillin/streptomycin and maintained at 37 °C in a humidified incubator with 10% CO_2_.

### Plasmids

The pcDNA3-FOXM1 has been described previously.^13^ The pmCherry-FOXM1 was generated by cloning the full-length FOXM1 cDNA from pcDNA3-FOXM1 into the *Eco*RI and *Bam*HI sites of the pmCherry-N1 vector (Clontech, Mountain View, CA, USA). The WT KIF20A and MUT KIF20A luciferase reporter constructs were generated from self-designed gene fragments which were synthesized by GeneArt (Life Technologies, Darmstadt, Germany) and cloned into the *Xho*I and *Bgl*lI sites of the pGL3-Basic vector (Promega, Madison, WI, USA). Expression plasmid transfections were performed with FuGENE 6 (Roche, Indianapolis, IN, USA) according to the manufacturer's recommendations.

### Luciferase reporter assay

MCF-7 cells were co-transfected with the human *KIF20A* luciferase reporter (WT or MUT), transfection control Renilla (pRL-TK; Promega, Southampton, UK) and pcDNA3-FOXM1 plasmids using FuGENE6 (Roche). For promoter analysis, 24 h after transfection, cells were collected, washed twice in phosphate-buffered saline and harvested for firefly/Renilla luciferase assays using the Dual-Glo Luciferase reporter assay system (Promega, Madison, WI, USA) according to the manufacturer's instruction. Luminescence was then read using the 9904 TOPCOUNT Perkin Elmer (Beaconsfield, UK) plate reader.

### Immunofluorescent staining

See Supplementary Materials and Methods

#### SAβ-gal assay

Cells were seeded in six-well plates at a density of approximately 20 000 cells/well before treatment with paclitaxel for 48 h. After culture for a further 5 days, cells were fixed and stained using a Senescence β-Galactosidase Staining Kit #9860 purchased from Cell Signalling Technology (Beverley, MA, USA). Plates were incubated overnight at 37 °C in a dry incubator (no CO_2_). Cells were then detected for blue staining under a bright-field microscope. The percentage of SAβ-gal-positive cells was calculated by counting the cells in five random fields.

#### Western blotting and antibodies

Western blotting was performed on whole-cell extracts by lysing cells in buffer as previously described.^11^ The antibodies against FOXM1 (C-20)(Cat#sc-502), β-tubulin (H-235) (Cat# sc-9104) and Cyclin B1 (Cat# sc-752) were purchased from Santa Cruz Biotechnology (Santa Cruz, CA, USA). The KIF20A antibody (ab104118) was purchased from Abcam (Cambridge, UK). The PARP (#9542) and Caspase7 (#9491) antibodies were purchased from Cell Signaling Technology (New England Biolabs Ltd., Hitchin, UK). Primary antibodies were detected using horseradish peroxidase-linked anti-mouse or anti-rabbit conjugates as appropriate (Dako, Glostrup, Denmark) and visualized using the ECL detection system (Amersham Biosciences, Pollards Wood, UK).

#### Quantitative real-time–PCR (qRT–PCR)

Total RNA was extracted with the RNeasy Mini Kit (Qiagen, Hilden, Germany). Complementary DNA generated by Superscript III reverse transcriptase and oligo-dT primers (Invitrogen, Paisley, UK) was analysed by qRT–PCR as described.^35^ Transcript levels were quantified using the standard curve method. The following gene-specific primers were used: L19-sense: 5′-GCGGAAGGGTACAGCCAAT-3′ and L19-antisense: 5′-GCAGCCGGCGCAAA-3′ FOXM1-sense: 5′-TCCTCCACCCCGAGCAA-3′ and FOXM1-antisense: 5′-CGTGAGCCTCCAGGATTCAG-3′ KIF20A-sense: 5′- GCCAACTTCATCCAACACCT -3′ and KIF20A -antisense: 5′- GTGGACAGCTCCTCCTCTTG -3′.

### Gene silencing with siRNAs

For gene silencing, cells were transiently transfected with siRNA SMARTpool reagents purchased from Thermo Scientific Dharmacon (Lafayette, CO, USA) using Oligofectamine (Invitrogen) according to the manufacturer's instructions. siRNAs used were: siRNA FOXM1 (L-009762-00), siRNA KIF20A (J-004957-06) and the NS (non-silencing) control siRNA, confirmed to have minimal targeting of known genes (D-001810-10-05).

### Clonogenic assay

See Supplementary Methods and Materials

### Cell cycle analysis

Cell cycle analysis was carried out by propidium iodide staining, as previously described.^13, 36^ The cell cycle profile was analysed using Cell Diva software (Becton Dickinson UK Ltd, Oxford, UK).

#### Chromatin immunoprecipitation (ChIP)

Cells were cross-linked in 1% formaldehyde, treated with glycine, scrapped and centrifuged. The pellets were washed with phosphate-buffered saline and resuspended in LB1 buffer (50 mM HEPES-KOH, pH 7.5; 140 mM NaCl; 1 mM EDTA; 10% glycerol; 0.5% Igepal CA-630; 0.25% Triton-X-100+1X PI). Samples were then lysed in LB2 buffer (10 mM Tris–HCl, pH 8; 200 mM NaCl; 1 mM EDTA; 0.5 mM EGTA+1X PI), centrifuged and resuspended in LB3 buffer (10 mM Tris-HCL, pH 8.0; 100 mM NaCl; 1 mM EDTA; 0.5 mM EGTA; 0.1% Na-Deoxycholate and 0.5% N-lauroylsarcosine+ 1X PI). DNA was fragmented to an average size of 150–200 bp using Bioruptor (Diagenode, Denville, NJ, USA). After sonication, Triton X-100 was added to a concentration of 1% and the mixture was centrifuged. Five percent of each sample was taken as input.

Twenty microlitres of Dynabeads conjugated to Protein A (Invitrogen) were pre-blocked by phosphate-buffered saline containing 0.5% bovine serum albumin and incubated with 4 μg of the indicated antibodies (anti-FOXM1 antibody (sc-502x, Santa Cruz) and rabbit IgG control (X0903, Dako). After overnight incubation with the antibodies, Dynabeads were washed twice and incubated with 100 μl of chromatin samples prepared previously for overnight at 4 °C. Dynabeads were subsequently washed in RIPA buffer and TE buffer before incubation overnight in elution buffer (1% SDS, 0.1 M NaHCO_3_) at 65 °C. After elution, supernatant was diluted with TE buffer and incubated with RNAse (Invitrogen) and proteinase K (ThermoFisher Scientific, Waltham, MA, USA), respectively. ChIP DNA was purified, dissovled in deionized water and subject to quantitative real-time PCR analysis. Data were presented as % Input using the following formula: % Input=100 × 2^(CT adjusted Input sample − CT immunoprecipitated sample). The experiments were repeated at least twice. The primers used are: 5′-TTCCTTACGCGGATTGGTAG-3′ (KIF20A sense) and 5′- AGCCGCAGAGCACAACTC-3′ (KIF20A anti-sense); 5′-CCGCCTCCCTCTTAGCATAA-3′ and 5′-CAGGAAATTGCATCTCGGGG-3′ (Control; -898/-724).

### Tissue microarray and immnohistochemistry

See Supplementary Methods and Materials

### Staining scoring

The stained tissue microarray slides were scanned by ScanScope scanners and individual stained tissue microarray spots were assessed in computer screen with the use of Aperio's image viewer, ImageScope. To avoid subjectivity in evaluation, the intensities and percentages of the staining were scored by two independent individuals in a semi-quantitative way as previously described and average was taken.^29, 41^ As KIF20A was detectable in both the cytoplasm and the nucleus, separate evaluation on cytoplasm and nucleus was carried out. For each case, a final score was obtained by multiplying the score of intensity with the score of percentage, 12 being the maximum final score. The total score is the sum of cytoplasm and nucleus scores.

### Statistical analysis

All statistics were determined using SPSS 16.0 and Windows XP, Excel (Imperial College, London, Software Shop, UK). The correlation between FOXM1 and KIF20A expression in tissue microarray was assessed by bi-variate Pearson Correlation analysis. The correlation between KIF20A expression and patients' survival was estimated by Kaplan–Meier estimation and compared by Log-rank test. Multivariate analysis was carried out by Cox-regression model. Protein and mRNA expression levels were compared by two-sided student *t*-tests.
