# Supplementary material for: Paclitaxel targets FOXM1 to regulate KIF20A in mitotic catastrophe and breast cancer paclitaxel resistance
Source: Oncogene. 2015 May 11;35(8):990–1002. doi: 10.1038/onc.2015.152 (PMC4538879; doi:10.1038/onc.2015.152)
Supplement: Supplementary Materials and Methods [file onc2015152x2.docx]

***Supplementary Materials and Methods***

***Clonogenic Assay***

A total 2,000 cells were seeded into six-well plates and incubated overnight. The cells were then treated for 48 h with varying concentrations of paclitaxel (Teva UK Limited, East Sussex, UK). The drug was removed and surviving cells were left to form colonies. After 14 days of incubation, colonies were fixed with 4% Paraformaldehyde for fifteen minutes at room temperature and then washed with phosphate buffered saline. 0.5% crystal violet was used to stain the fixed cells for thirty minutes, following which the plates were washed with tap water. Plates were then left to dry overnight. Quantification was achieved by solubilising dye with 33% acetic acid and the absorbance was measured at 492nm using a microplate reader (Sunrise, Tecan).

***Tissue Microarray***

One hundred and thirty-three cases of breast cancer diagnosed between the years 1992 to 2001 with clinical follow up data were retrieved from the records of the Department of Pathology, Queen Mary Hospital of Hong Kong, with approval by the Institutional Review Board of The University of Hong Kong with patient consents. Histological sections of all cases were reviewed by the pathologist, the representative paraffin tumour blocks chosen as donor block for each case and the selected areas marked for construction of tissue microarray (TMA) blocks. A total of 116 could be assessed and scored for FOXM1 and KIF20A staining. The expression pattern and subcellular localization were correlated with various clinicopathological data including ER, PR status, age, histological grade, histological type, clinical stage, lymph node metastasis as well as survival time.

***Immnohistochemistry***

The TMA sections were deparaffinized and rehydrated by incubation with xylene and decreasing concentrations of ethanol. Citrate buffer (0.01M, pH 6.0) was used for antigen retrieval. The slides were immersed into 3% H_2_O_2_/methanol for 10 min at room temperature to quench endogenous peroxidase. After rinsing in 0.05% Tween in PBS (PBST) twice, FOXM1 or KIF20A specific antibody diluted at 1:1100 or 1:50 (Abcam, Cambridge, UK) was added to each section and incubated at 4°C overnight. The slides were then washed in PBST and incubated with DAKO EnVision+System-HRP-labelled Polymer Anti-Rabbit at room temperature in dark for 30 min. After washing, Chromogen DAB/substrate reagent was added onto the slides and the slides incubated for a further 6 minutes. Finally, the slides were dehydrated and mounted. Aperio ScanScope ® system (Aperio technology, USA) was used to visualize and assess for FOXM1 and KIF20A expression.

***Immunofluorescent staining***

Cells grown on chamber culture slides were fixed in 4% paraformaldehyde (Thermo Scientific, Rockford, IL, USA) for 15 min followed by permeabilization for 10 min with 0.2% Triton X-100 in PBS, and blocking with 5% goat serum for 30 min at room temperature. The slides were incubated overnight at 4 °C with primary antibodies, anti-α-tubulin (clone DM1A) and anti-γ-tubulin purchased from Sigma-Aldrich (St. Louis, MO, USA). The slides were then rinsed with PBS and subsequently incubated with a 1:500 dilution of Alexa Fluor 488-conjugated goat anti-mouse and Alexa Fluor 555-conjugated goat anti-rabbit secondary antibodies (Molecular Probes, Invitrogen) for 45 min at room temperature. After washing with PBS, cells were mounted with Vectashield mounting solution containing DAPI (Vector Laboratories). Mitotic cells were visualized with a Leica TCS SP5 confocal microscope (Leica Microsystems, Mannhein, Germany) equipped with a 63x oil immersion objective and LAS-AF software. For each condition, images of at least 50 mitotic cells were captured and analysed.
