# Supplementary material for: Paclitaxel targets FOXM1 to regulate KIF20A in mitotic catastrophe and breast cancer paclitaxel resistance
Source: Oncogene. 2015 May 11;35(8):990–1002. doi: 10.1038/onc.2015.152 (PMC4538879; doi:10.1038/onc.2015.152)
Supplement: Supplementary Figure Legends [file onc2015152x3.docx]

**Supplementary Figure Legends**

**Supplementary Figure S1. Paclitaxel resistant MCF-7 cells exhibit up-regulated expression levels of FOXM1 and KIF20A. (A)** Protein expression levels of FOXM1, KIF20A, cyclin B1 and PARP in MCF-7 and MCF-7Tax^R^ cell lines were examined by western blotting after paclitaxel treatment at different time points indicated (a repeat of Fig. 2A). Notably, there was no cleavage of PARP. **(B)** Densitometric analysis of the western blots in **(A)** was carried out using Image J software. The intensity of the bands of FOXM1 and KIF20A at each time point was normalised to the intensity of the β-Tubulin band at the respective time point and the obtained values were plotted against time after paclitaxel. **(C)** Densitometric analysis of the western blots in **(Fig. 2A)** was carried out using Image J software. The intensity of the bands of FOXM1 and KIF20A at each time point was normalised to the intensity of the β-Tubulin band at the respective time point and the obtained values were plotted against time after paclitaxel. The relative expression levels of FOXM1 and KIF20A show similar kinetics in MCF-7 and MCF-7Tax^R^ cell lines following paclitaxel treatment in (A) and (B).

**Supplementary Figure S2. FOXM1 depletion decreases KIF20A expression in MCF-7 and MCF-7Tax^R^ breast cancer cells. (A)** MCF-7 and MCF-7 Tax^R^ cells were transfected with Smart pool siRNA against FOXM1 or with non-silencing controls (NSC). FOXM1 and KIF20A mRNA levels were determined by qRT-PCR (a repeat of Fig. 2B). Bars represent mean±S.D. Statistical significance was determined by Student’s t-test, 2-sided **P≤0.01, , significant; n.s., non-significant). **(B)** The expression levels of FOXM1, KIF20A and β-tubulin were analysed by western blot analysis (Fig. 2B). Densitometric analysis of the western blots in **(Fig. 2A)** was carried out using Image J software. The intensity of the bands of FOXM1 and KIF20A at each time point was normalised to that of the β-Tubulin band at the respective time point and the obtained values plotted against time after epirubicin. **(C)** The expression levels of FOXM1, KIF20A and β-tubulin were analysed by western blot analysis (a repeat of Fig. 2C).

**Supplementary Figure S3. FOXM1 depletion decreases KIF20A expression in MDA-MB-231 breast cancer cells.** MDA-MB-231 breast cancer cells were transfected with Smart pool siRNA against FOXM1 or with non-silencing controls (NSC). (A) FOXM1 and KIF20A mRNA levels were determined by RT-qPCR. Bars represent mean±S.D. Statistical significance was determined by Student’s t-test, 2-sided (**P≤0.01, ***P≤0.005, significant; n.s., non-significant). (B) The expression levels of FOXM1, KIF20A and β-tubulin were analysed by western blot analysis.

**Supplementary Figure S4. FOXM1 fails to *trans*activate a putative *KIF20A* promoter region containing the 5’ UTR in the MCF-7 breast cancer cells**

Schematic representation of a putative *KIF20A* promoter region containing the 5’ UTR (Upper panel). MCF-7 cells were transiently co-transfected with the pGL3 control or the pGL3 basic luciferase reporter plasmid containing the putative *KIF20A* promoter, the Renilla luciferase plasmid and the FOXM1 expression plasmid as indicated. Twenty-four hours after transfection, cells were lysed, and the luciferase activity examined. Firefly luminescence signal was normalized with the Renilla luminescence signal. Bars represent mean±S.D (n=3).

**Supplementary Figure S5. MCF-7 cells does not undergo apoptotic PARP and Caspase 7-cleavage associated apoptotic cell death following paclitaxel treatment and/or FOXM1 or KIF20A depletion.**

MCF-7 cells were transfected with NSC, FOXM1 or KIF20A siRNA. 24 h after transfection, cells were treated with 10 nM paclitaxel for 0, 24 and 48 h. The cells were then harvested for western blot analysis. The expression levels of FOXM1, KIF20A, PARP, Caspase 7 and β-tubulin were analysed by western blot analysis. The results showed that neither paclitaxel treatment nor FOXM1 or KIF20A depletion induces apoptosis-associated PARP and Caspase 7-cleavage.

**Supplementary Figure S6. Increased in MCF-7 cells with lagging chromosomes in anaphase following FOXM1 or KIF20A depletion.** MCF-7 cells were transfected with NSC, FOXM1 or KIF20A siRNA. Twenty-four h after transfection, cells were then fixed, permeabilized, and immunostained with antibody against α-tubulin (Green) and γ-tubulin (Red). Nuclei were counterstained with DAPI (Blue). Mitotic cells were visualized with Leica TCS SP5 (63 × magnification). For each condition, images of at least 50 anaphase cells were captured. Representative confocal images are shown (upper panel). The number of anaphase cells with lagging chromosomes was quantified. Results represent average of three independent experiments ± S.D. Statistical significance was determined by Student’s t-test, 2-sided (not significant, ns; very significant **P ≤ 0.01).

**Supplementary Figure S7. Accumulation of MCF-7 cells displaying mitotic catastrophe phenotype following paclitaxel treatment or FOXM1 or KIF20A depletion.** MCF-7 cells were either transfected with NSC, FOXM1 or KIF20A siRNA or treated with 5nM paclitaxel. Twenty-four h after transfection or incubation with paclitaxel, cells were then fixed, permeabilized, and immunostained with antibody against α-tubulin (Green) and γ-tubulin (Red). Nuclei were counterstained with DAPI (Blue). Cells were visualized with Leica TCS SP5 (63 × magnification). Representative confocal images are shown. Cells treated with paclitaxel or transfected with either FOXM1 or KIF20A showed mitotic catastrophe morphologies of multi or micro-nucleation.

**Supplementary Figure S8. Depletion of KIF20A or FOXM1 inhibits cell proliferation and induces senescence in MCF-7 and MCF-7Tax^R^ breast cancer cells.** MCF-7 and MCF-7Tax^R^ cells were transfected with either NSC (non-silencing control) siRNA, siRNA targeting FOXM1 or KIF20A. Twenty-four hours after transfection, 2,000 cells were seeded in six well plates, treated with 0, 1, 3, 5, 7.5 and 10 nM of paclitaxel, grown for 15 days and then stained with crystal violet (Top). The result (Bottom) represents average of three independent experiments ± SD. Statistical significance was determined by Student’s t-test, 2-sided (*P≤0.05, **P≤0.01, ***P ≤0.005; n.s., non-significant).

**Supplementary Figure** **S9.** **Transfection efficiency of FOXM1 siRNA and KIF20A siRNA in MCF-7 cells.** **(A)** Transfection efficiency was monitored by the uptake of the BLOCK-iT fluorescent oligo (green fluorescence) at 48 h following transfection using Oligofectamine. More than 70% of cells were successfully transfected. **(B)** FOXM1 and KIF20A mRNA expression levels were detected by real-time qPCR at 48 h post-transfection. Bars represent average±S.D. Statistical significance was determined by Student’s t-test, 2-sided (**P≤0.01). **(C)** FOXM1 and KIF20A protein expression levels were detected by Western blot analysis at 48 h post-transfection.

**Supplementary Figure S10.** **Overexpression of FOXM1 confers paclitaxel resistance.** MCF-7 cells were transfected with control empty vector or pcDNA3-FOXM1 plasmid. Cells were then treated with increasing concentrations of paclitaxel for 48 h and their cell viability measured by sulforhodamine B (SRB) assay (Top panel). Representative data from three independent experiments are shown. Statistical analyses were performed using Student’s t-tests, 2-sided (**P≤0.05) (n=3). Western blot analysis of FOXM1 and β-Tubulin expression in the transfected cells.

**Supplementary Figure** **S11.** **Transfection efficiency of FOXM1 siRNA and KIF20A siRNA in MCF-7Tax^R^ cells.** **(A)** Transfection efficiency was monitored by the uptake of the BLOCK-iT fluorescent oligo (green fluorescence) at 48 h following transfection using Oligofectamine. More than 70% of cells were successfully transfected. **(B)** FOXM1 and KIF20A mRNA expression levels were detected by real-time qPCR at 48 h post-transfection. Bars represent average±S.D (n=3). Statistical significance was determined by Student’s t-test, 2-sided (**P≤0.01). **(C)** FOXM1 and KIF20A protein expression levels were detected by Western blot analysis at 48 h post-transfection.

**.**

**Supplementary Figure S12.** **Univarite and multivariate Cox-regression analysis using KIF20A nuclear score and other clinicopathological parameters**. Nuclear KIF20A overexpression is associated with higher risk of death in univariate analysis (*P* = 0.05, RR=2.141 for overall survival and *P*=0.02, RR=2.695 for disease-specific survival, respectively) and remained associated with poor survival after correcting for tumour stage and lymph-node involvement (*P* = 0.047, RR=2.47 for overall survival and *P*=0.037, RR=2.767 for disease-specific survival, respectively)

**Supplementary Figure S13.** **Nuclear KIF20A over-expression significantly associated with poorer survival in breast cancer patients who had received chemotherapy** (log-rank test, *P* = 0.008 for overall survival and *P*=0.004 for disease-specific survival, respectively). In this cohort, Cox-regression analysis showed that nuclear KIF20A over-expression is an even stronger risk marker (*P*=0.013, RR=4.008 for overall survival and *P*=0.01, RR=5.089 for disease-specific survival, respectively).
