# Supplementary material for: Paclitaxel targets FOXM1 to regulate KIF20A in mitotic catastrophe and breast cancer paclitaxel resistance
Source: Oncogene. 2015 May 11;35(8):990–1002. doi: 10.1038/onc.2015.152 (PMC4538879; doi:10.1038/onc.2015.152)
Supplement: Supplementary Figure 1 [file onc2015152x4.ppt]

## Slide 1
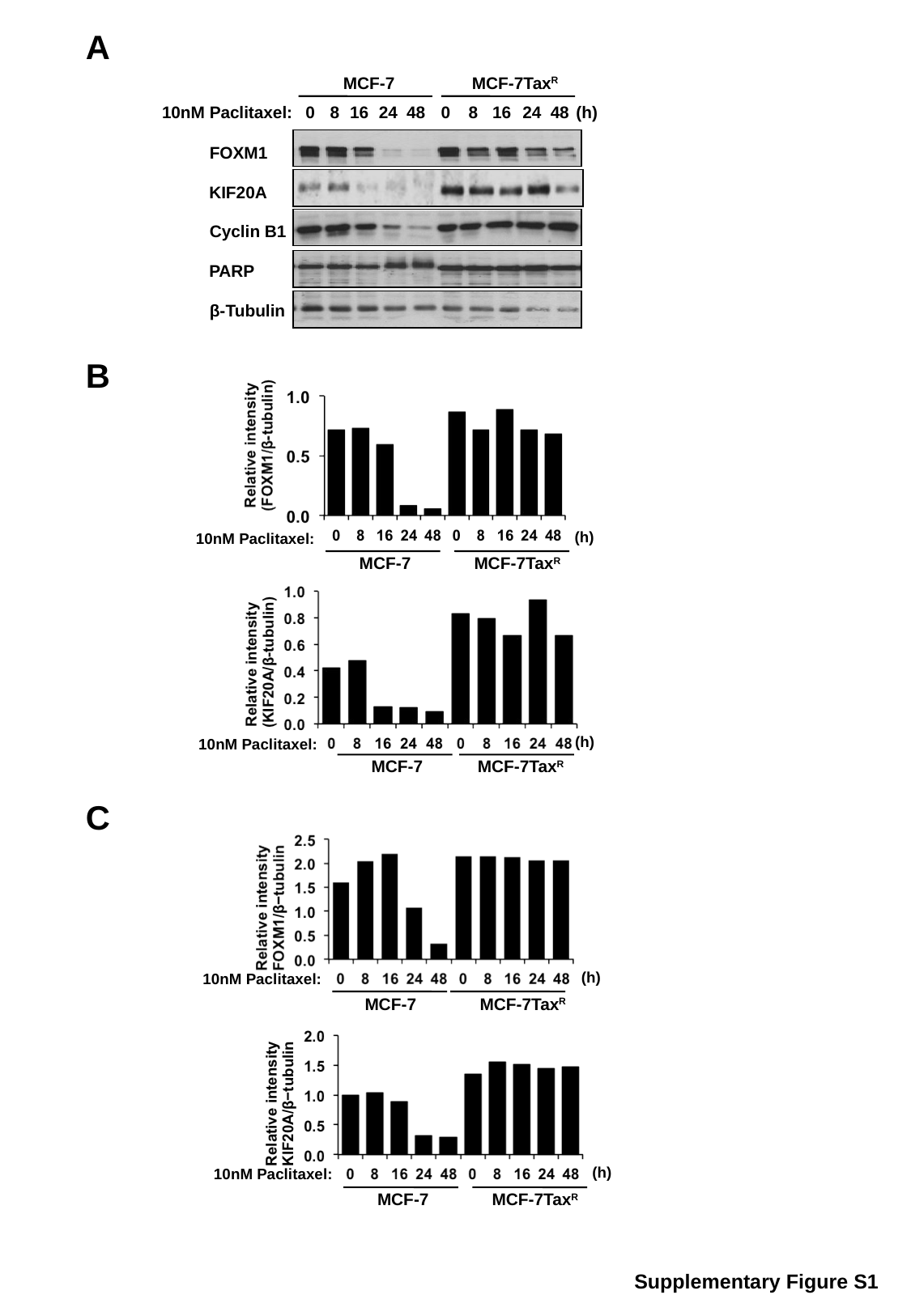

A
MCF-7TaxR
MCF-7
10nM Paclitaxel:
0
8
16
24
48
0
8
16
24
48
(h)
FOXM1
KIF20A
Cyclin B1
PARP
β-Tubulin
B
(h)
10nM Paclitaxel:
MCF-7
MCF-7TaxR
(h)
10nM Paclitaxel:
MCF-7
MCF-7TaxR
C
(h)
10nM Paclitaxel:
MCF-7
MCF-7TaxR
(h)
10nM Paclitaxel:
MCF-7
MCF-7TaxR
Supplementary Figure S1
