# Supplementary material for: Paclitaxel targets FOXM1 to regulate KIF20A in mitotic catastrophe and breast cancer paclitaxel resistance
Source: Oncogene. 2015 May 11;35(8):990–1002. doi: 10.1038/onc.2015.152 (PMC4538879; doi:10.1038/onc.2015.152)
Supplement: Supplementary Figure 2 [file onc2015152x5.ppt]

## Slide 1
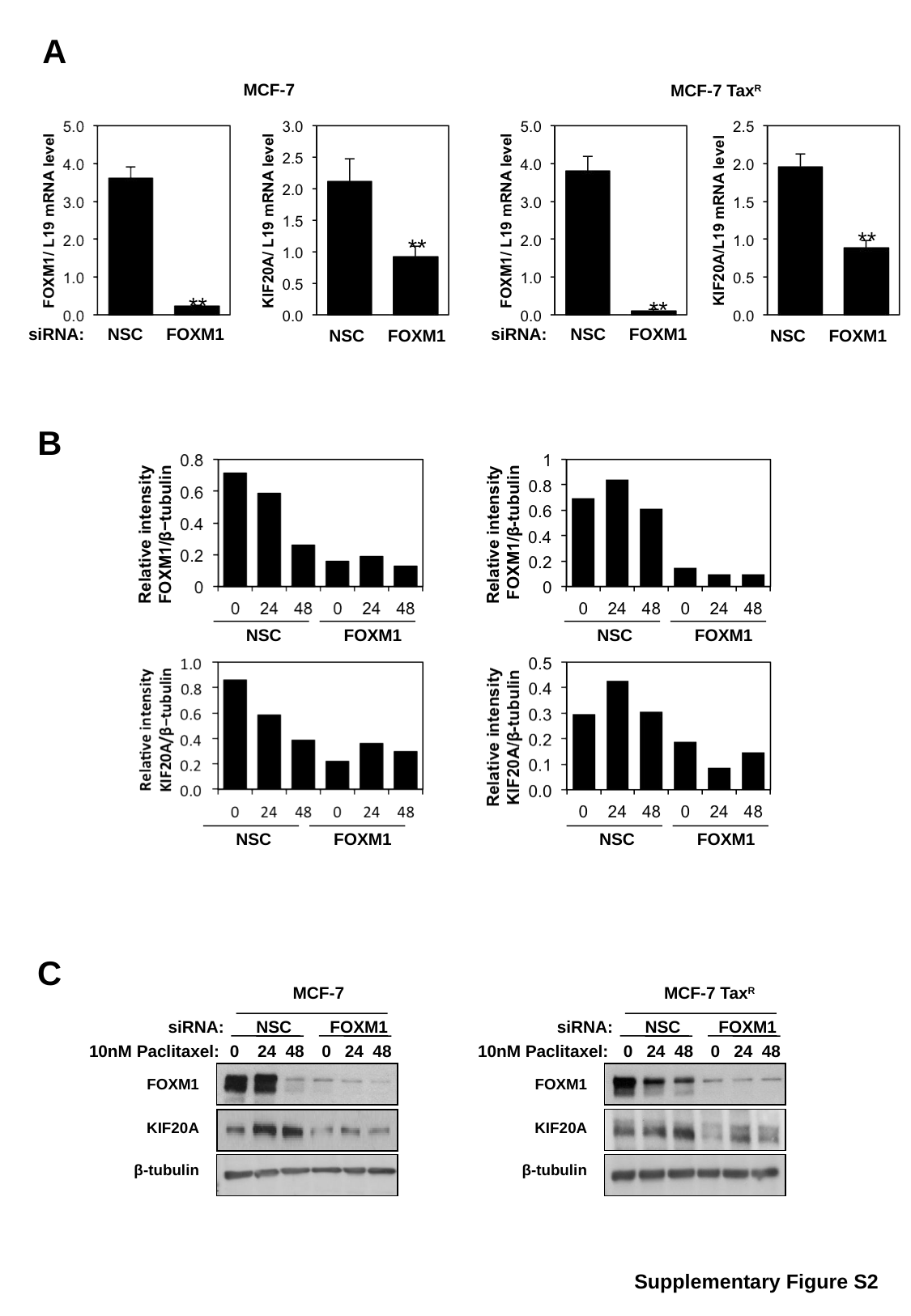

A
MCF-7
MCF-7 TaxR
**
**
**
**
 siRNA: NSC FOXM1
 siRNA: NSC FOXM1
 NSC FOXM1
 NSC FOXM1
B
NSC
FOXM1
NSC
FOXM1
NSC
FOXM1
NSC
FOXM1
C
MCF-7
siRNA:
NSC
FOXM1
0 24 48
 0 24 48
10nM Paclitaxel:
MCF-7 TaxR
siRNA:
NSC
FOXM1
 0 24 48
 0 24 48
10nM Paclitaxel:
FOXM1
FOXM1
KIF20A
KIF20A
β-tubulin
β-tubulin
Supplementary Figure S2
