# Supplementary figures and images for: Paclitaxel targets FOXM1 to regulate KIF20A in mitotic catastrophe and breast cancer paclitaxel resistance
Source: Oncogene. 2015 May 11;35(8):990–1002. doi: 10.1038/onc.2015.152 (PMC4538879; doi:10.1038/onc.2015.152)

## Slide 1
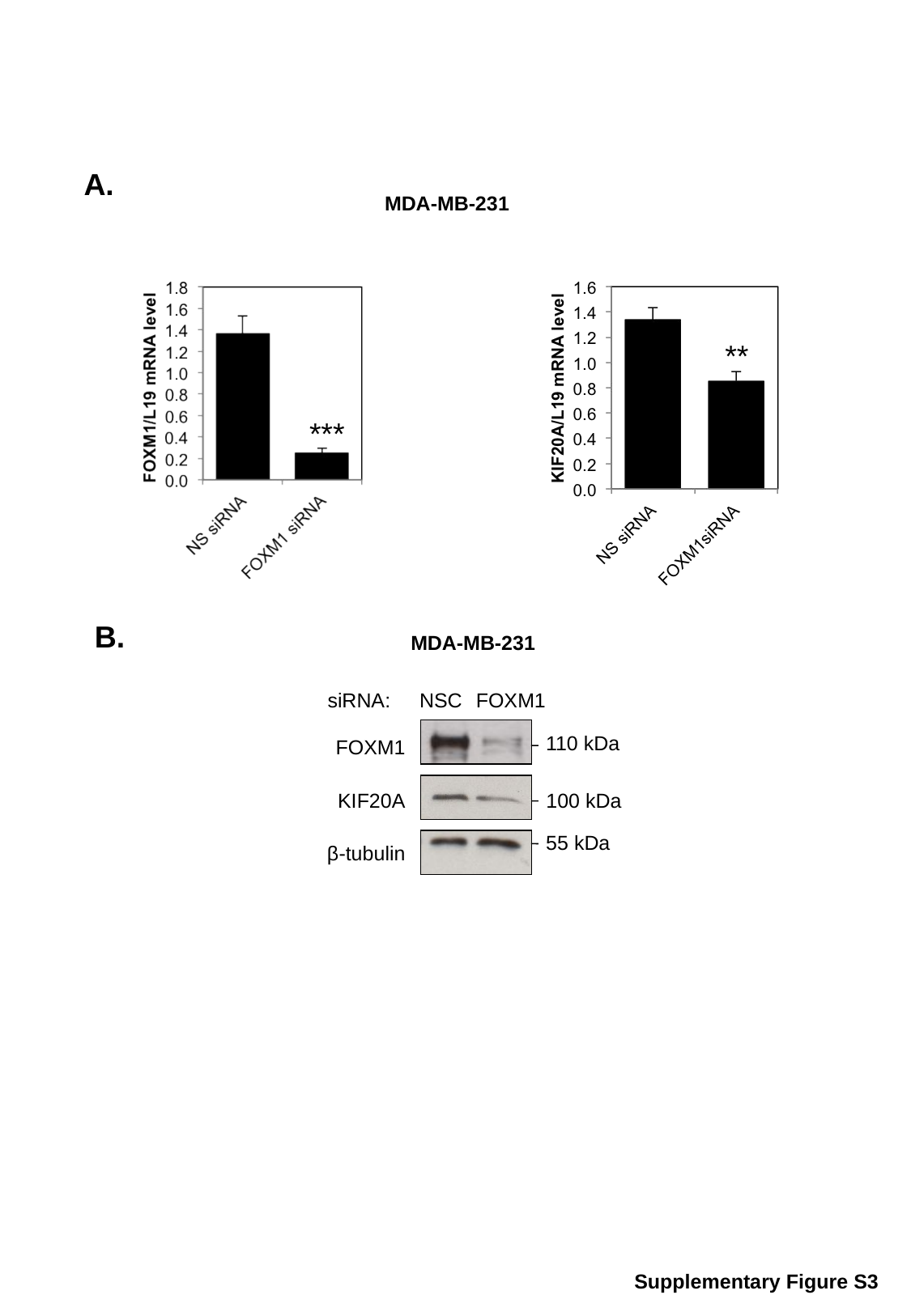

A.
MDA-MB-231
**
***
B.
MDA-MB-231
siRNA:
NSC
FOXM1
110 kDa
FOXM1
KIF20A
100 kDa
55 kDa
β-tubulin
Supplementary Figure S3

Supplement: Supplementary Figure 3 [file onc2015152x6.ppt]

## Slide 1
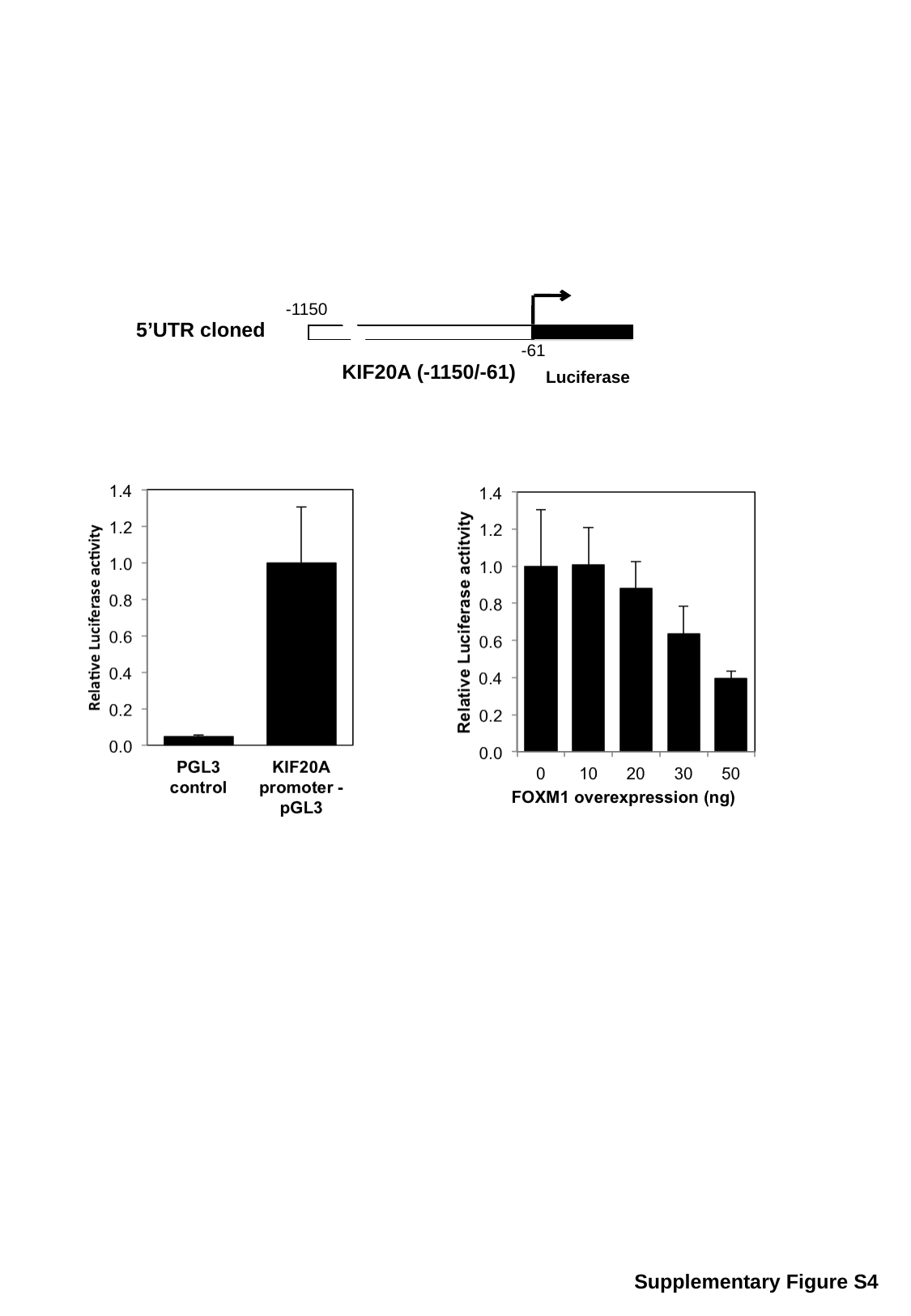

-1150
5’UTR cloned
-61
KIF20A (-1150/-61)
Luciferase
Supplementary Figure S4
Supplementary Figure S2

Supplement: Supplementary Figure 4 [file onc2015152x7.ppt]

## Slide 1
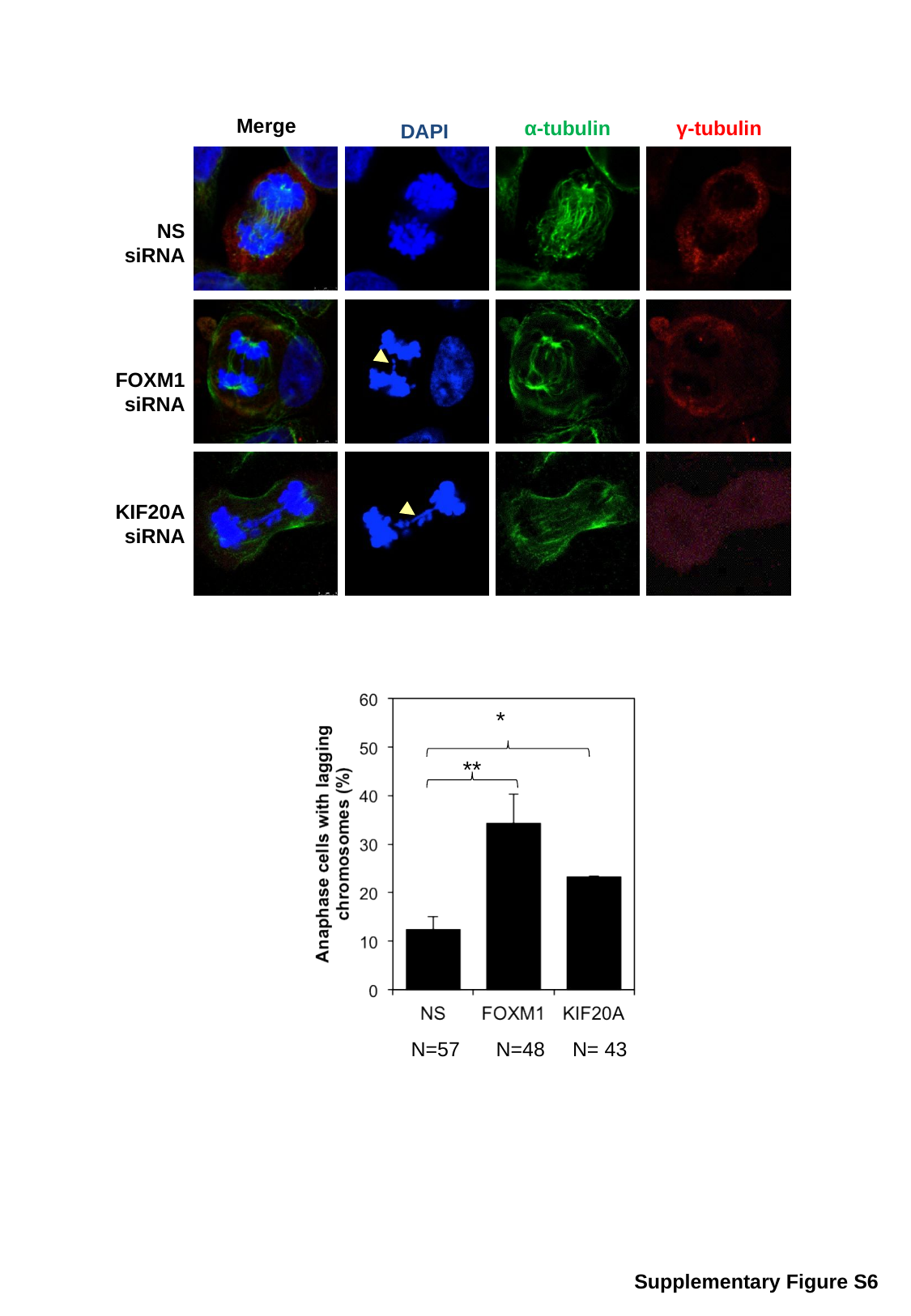

Merge
 α-tubulin
γ-tubulin
 DAPI
NS
siRNA
FOXM1
siRNA
KIF20A
siRNA
*
**
N=57
N=48
N= 43
Supplementary Figure S6

Supplement: Supplementary Figure 6 [file onc2015152x9.ppt]

## Slide 1
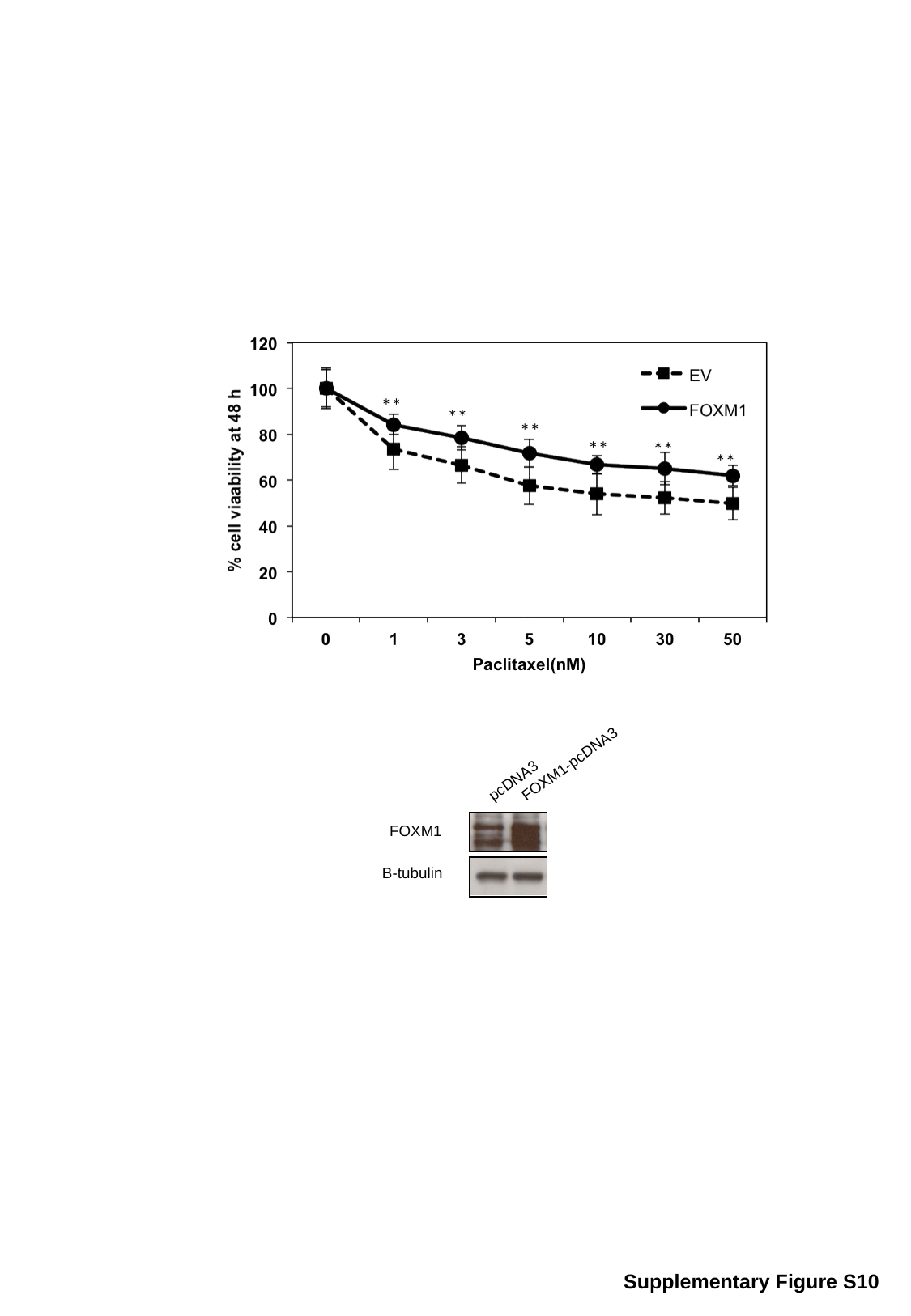

FOXM1-pcDNA3
pcDNA3
FOXM1
Β-tubulin
**
**
**
**
**
**
Supplementary Figure S10

Supplement: Supplementary Figure 10 [file onc2015152x13.ppt]
