# Supplementary material for: Paclitaxel targets FOXM1 to regulate KIF20A in mitotic catastrophe and breast cancer paclitaxel resistance
Source: Oncogene. 2015 May 11;35(8):990–1002. doi: 10.1038/onc.2015.152 (PMC4538879; doi:10.1038/onc.2015.152)
Supplement: Supplementary Figure 5 [file onc2015152x8.ppt]

## Slide 1
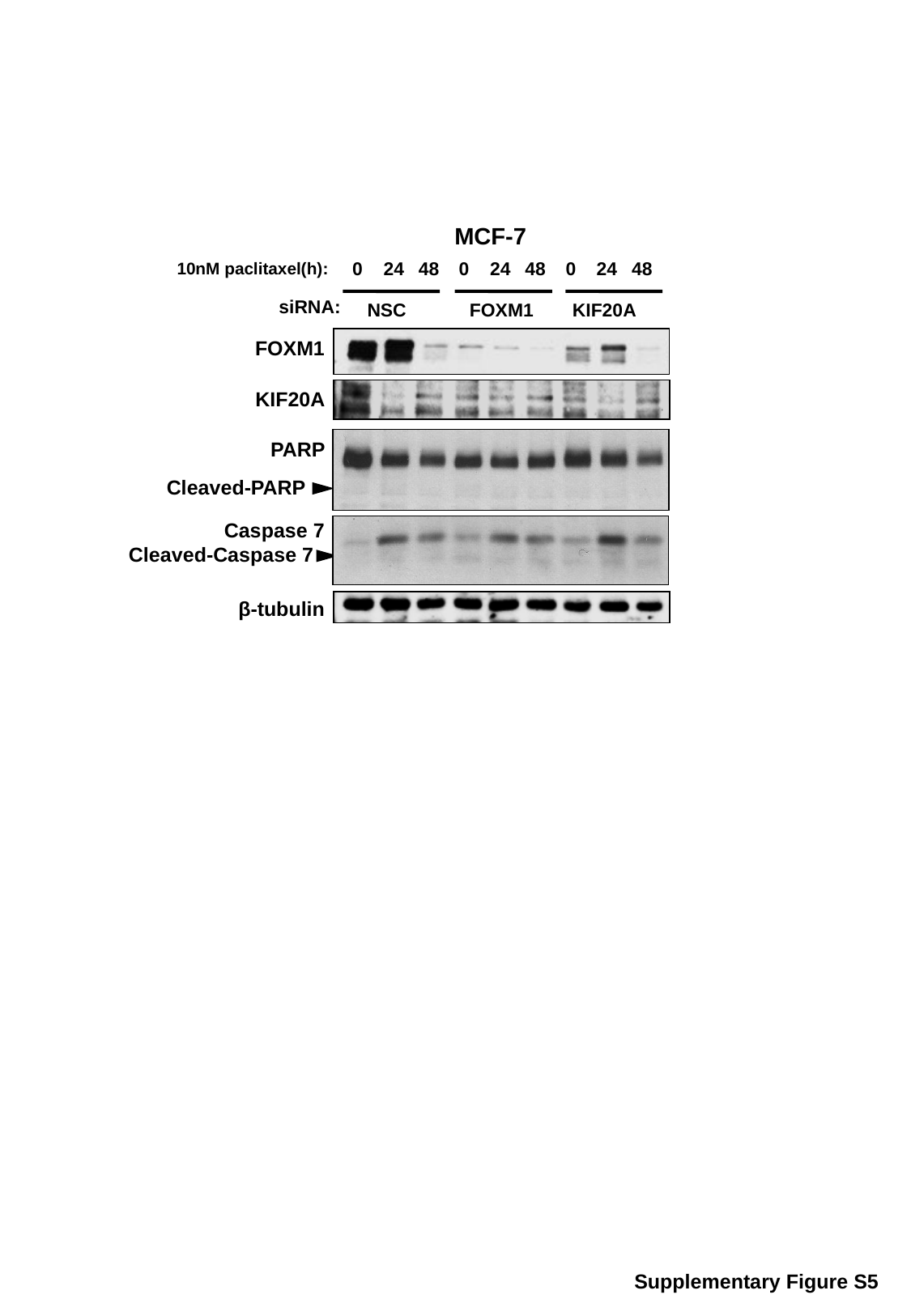

MCF-7
0
24
48
0
24
48
0
24
48
10nM paclitaxel(h):
siRNA:
NSC
FOXM1
KIF20A
FOXM1
KIF20A
PARP
Cleaved-PARP
Caspase 7
Cleaved-Caspase 7
β-tubulin
Supplementary Figure S5
