# Supplementary material for: Paclitaxel targets FOXM1 to regulate KIF20A in mitotic catastrophe and breast cancer paclitaxel resistance
Source: Oncogene. 2015 May 11;35(8):990–1002. doi: 10.1038/onc.2015.152 (PMC4538879; doi:10.1038/onc.2015.152)
Supplement: Supplementary Figure 7 [file onc2015152x10.ppt]

## Slide 1
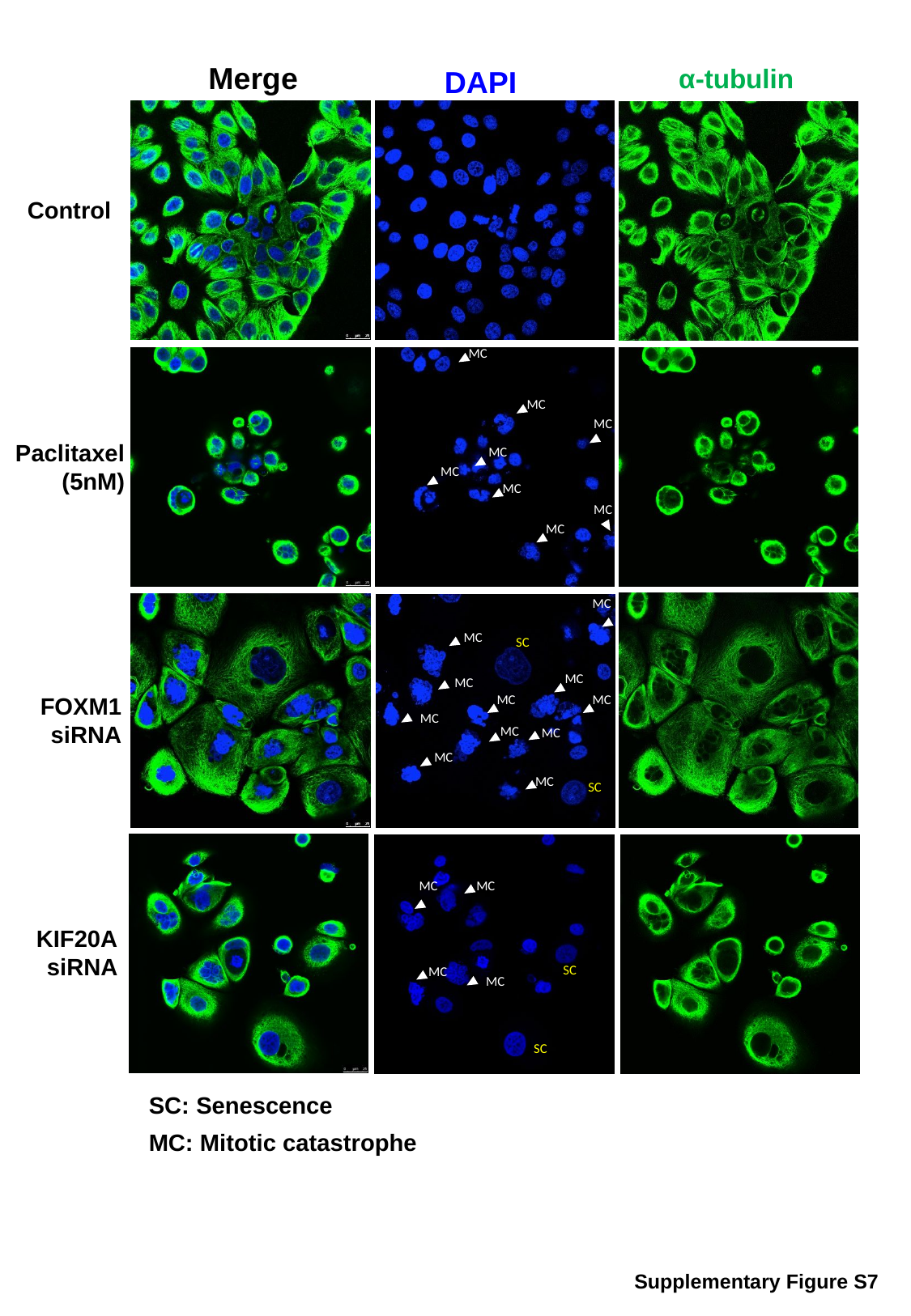

Merge
 α-tubulin
DAPI
Control
MC
MC
MC
Paclitaxel
(5nM)
MC
MC
MC
MC
MC
MC
MC
SC
MC
MC
MC
MC
MC
MC
MC
MC
MC
SC
FOXM1
siRNA
MC
MC
SC
MC
MC
SC
KIF20A
siRNA
SC: Senescence
MC: Mitotic catastrophe
Supplementary Figure S7
