# Supplementary material for: Paclitaxel targets FOXM1 to regulate KIF20A in mitotic catastrophe and breast cancer paclitaxel resistance
Source: Oncogene. 2015 May 11;35(8):990–1002. doi: 10.1038/onc.2015.152 (PMC4538879; doi:10.1038/onc.2015.152)
Supplement: Supplementary Figure 8 [file onc2015152x11.ppt]

## Slide 1
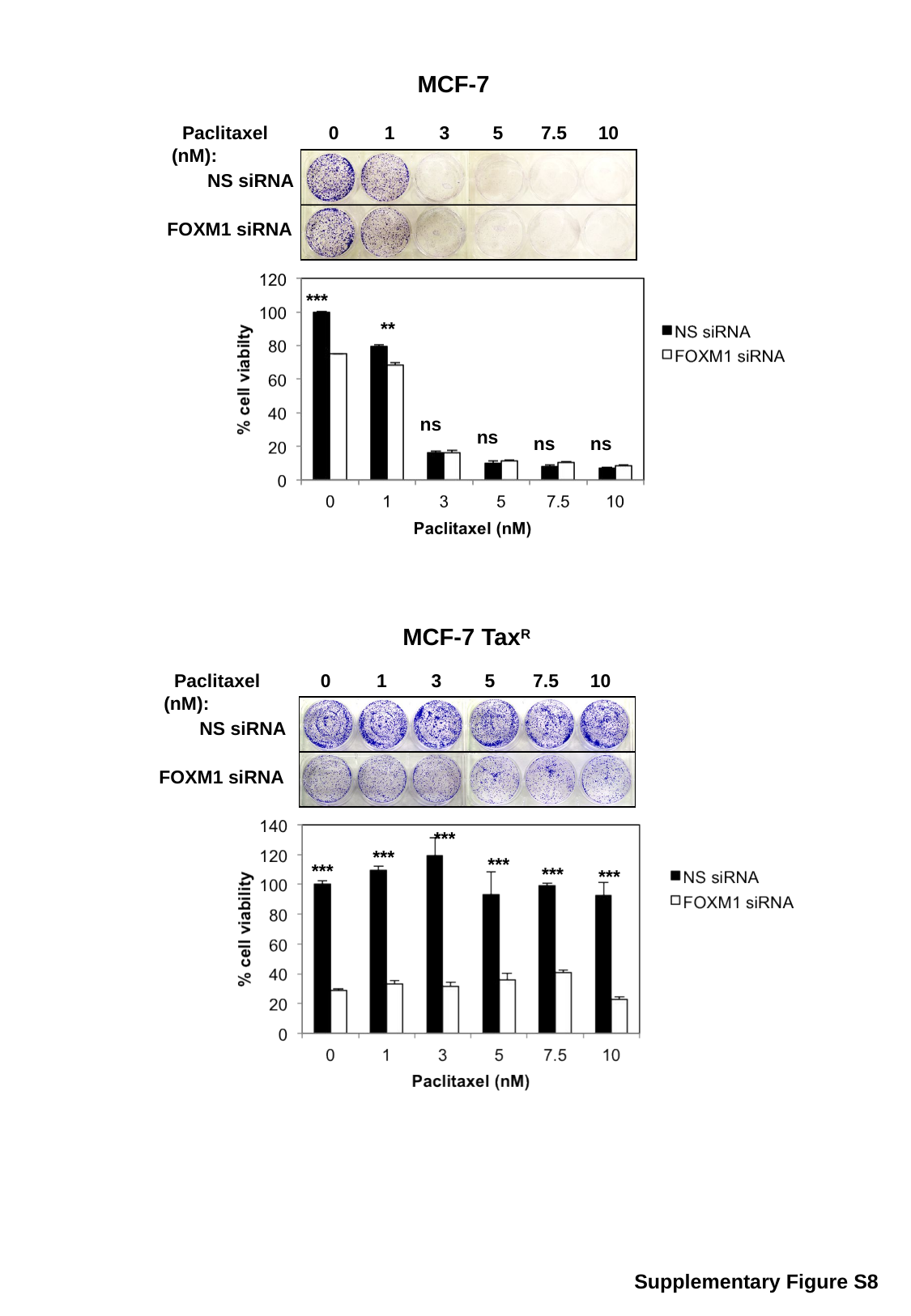

MCF-7
 Paclitaxel (nM):
0
1
3
5
7.5
10
NS siRNA
FOXM1 siRNA
***
**
ns
ns
ns
ns
MCF-7 TaxR
 Paclitaxel (nM):
0
1
3
5
7.5
10
NS siRNA
FOXM1 siRNA
***
***
***
***
***
***
Supplementary Figure S8
