# Supplementary material for: Paclitaxel targets FOXM1 to regulate KIF20A in mitotic catastrophe and breast cancer paclitaxel resistance
Source: Oncogene. 2015 May 11;35(8):990–1002. doi: 10.1038/onc.2015.152 (PMC4538879; doi:10.1038/onc.2015.152)
Supplement: Supplementary Figure 9 [file onc2015152x12.ppt]

## Slide 1
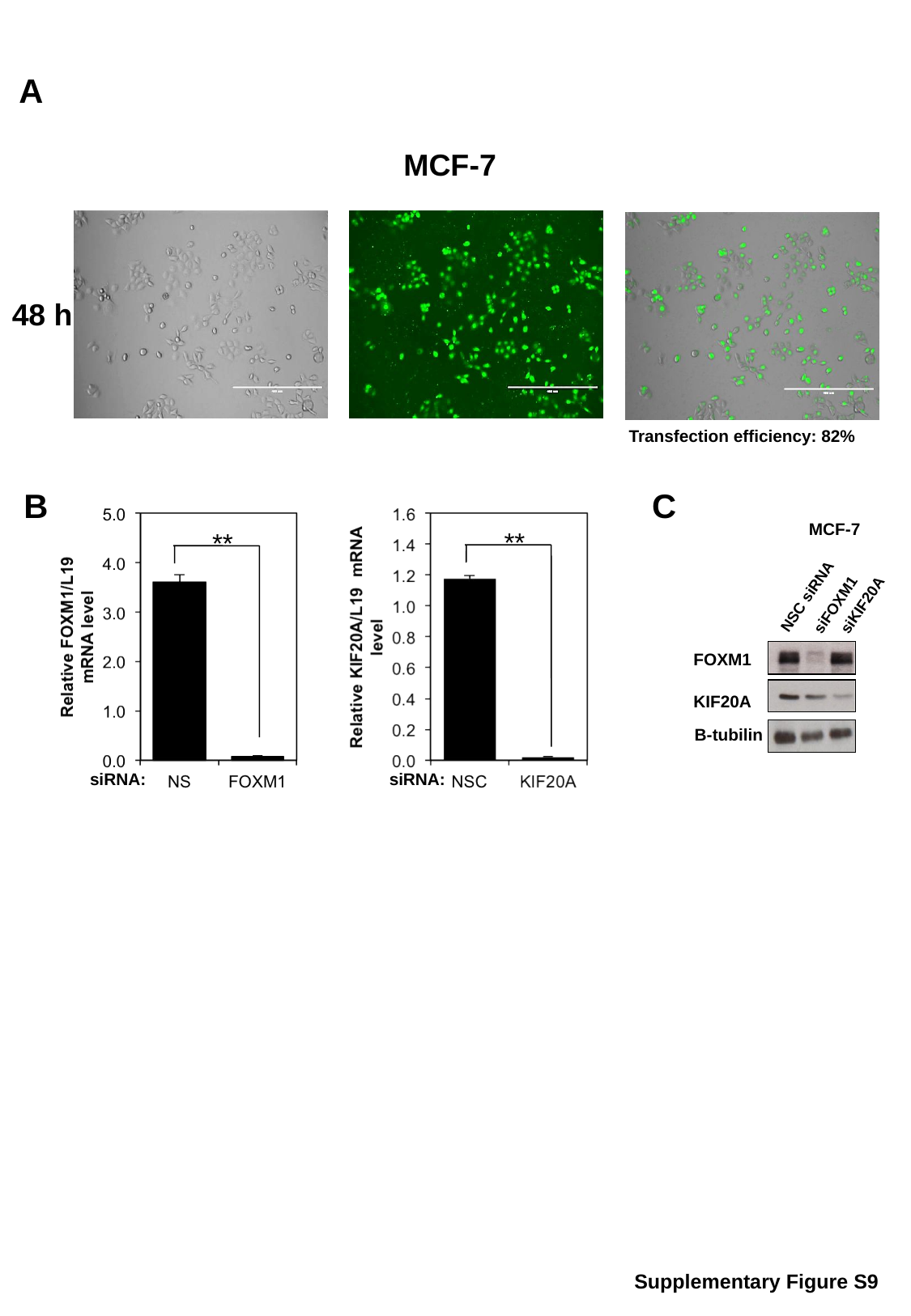

A
MCF-7
48 h
Transfection efficiency: 82%
B
C
MCF-7
**
**
NSC siRNA
siFOXM1
siKIF20A
FOXM1
KIF20A
Β-tubilin
siRNA:
siRNA:
Supplementary Figure S9
