# Supplementary material for: Paclitaxel targets FOXM1 to regulate KIF20A in mitotic catastrophe and breast cancer paclitaxel resistance
Source: Oncogene. 2015 May 11;35(8):990–1002. doi: 10.1038/onc.2015.152 (PMC4538879; doi:10.1038/onc.2015.152)
Supplement: Supplementary Figure 11 [file onc2015152x14.ppt]

## Slide 1
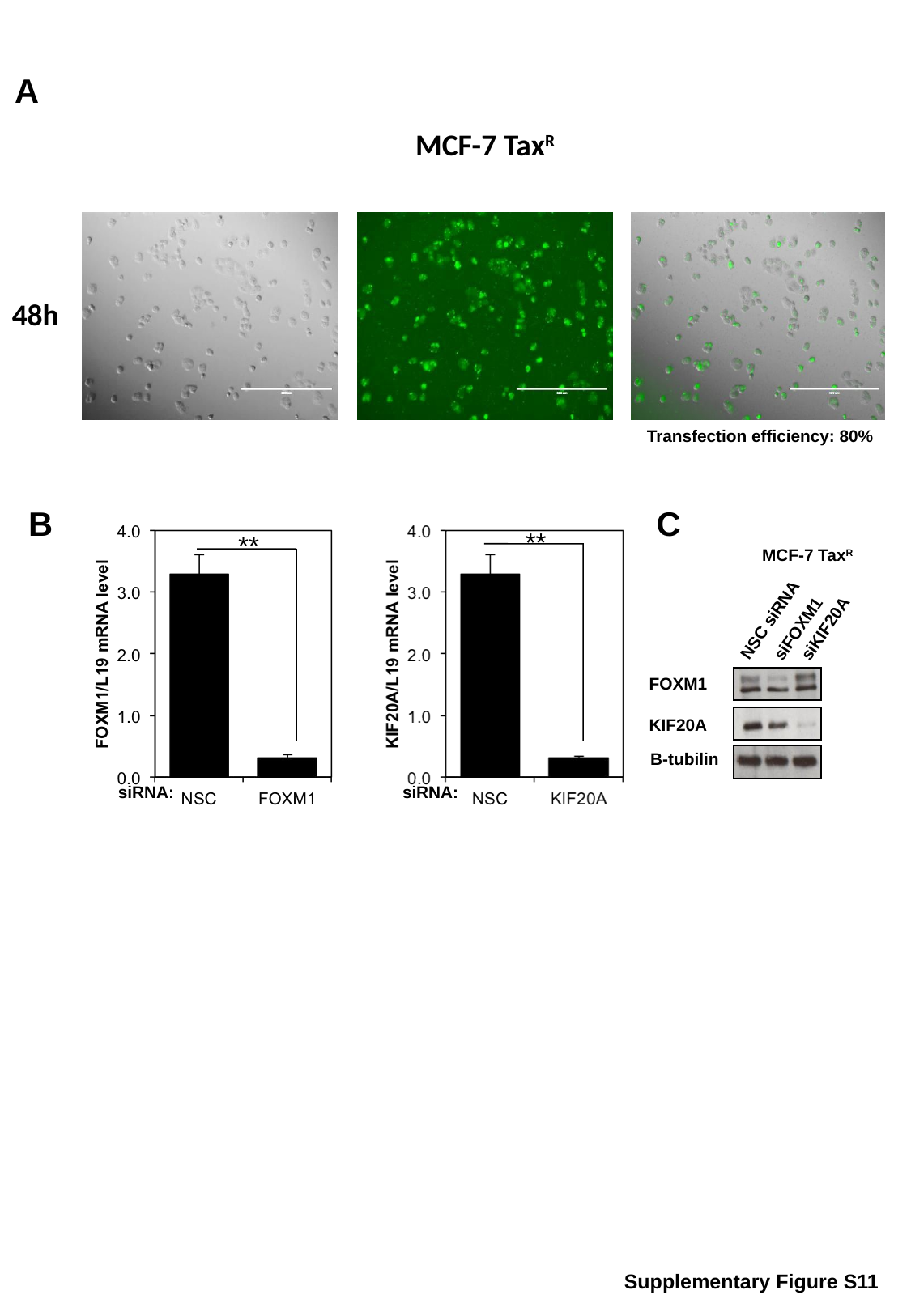

A
MCF-7 TaxR
48h
Transfection efficiency: 80%
B
C
**
**
MCF-7 TaxR
NSC siRNA
siKIF20A
siFOXM1
FOXM1
KIF20A
Β-tubilin
siRNA:
siRNA:
Supplementary Figure S11
