# Supplementary material for: Paclitaxel targets FOXM1 to regulate KIF20A in mitotic catastrophe and breast cancer paclitaxel resistance
Source: Oncogene. 2015 May 11;35(8):990–1002. doi: 10.1038/onc.2015.152 (PMC4538879; doi:10.1038/onc.2015.152)
Supplement: Supplementary Figure 12 [file onc2015152x15.ppt]

## Slide 1
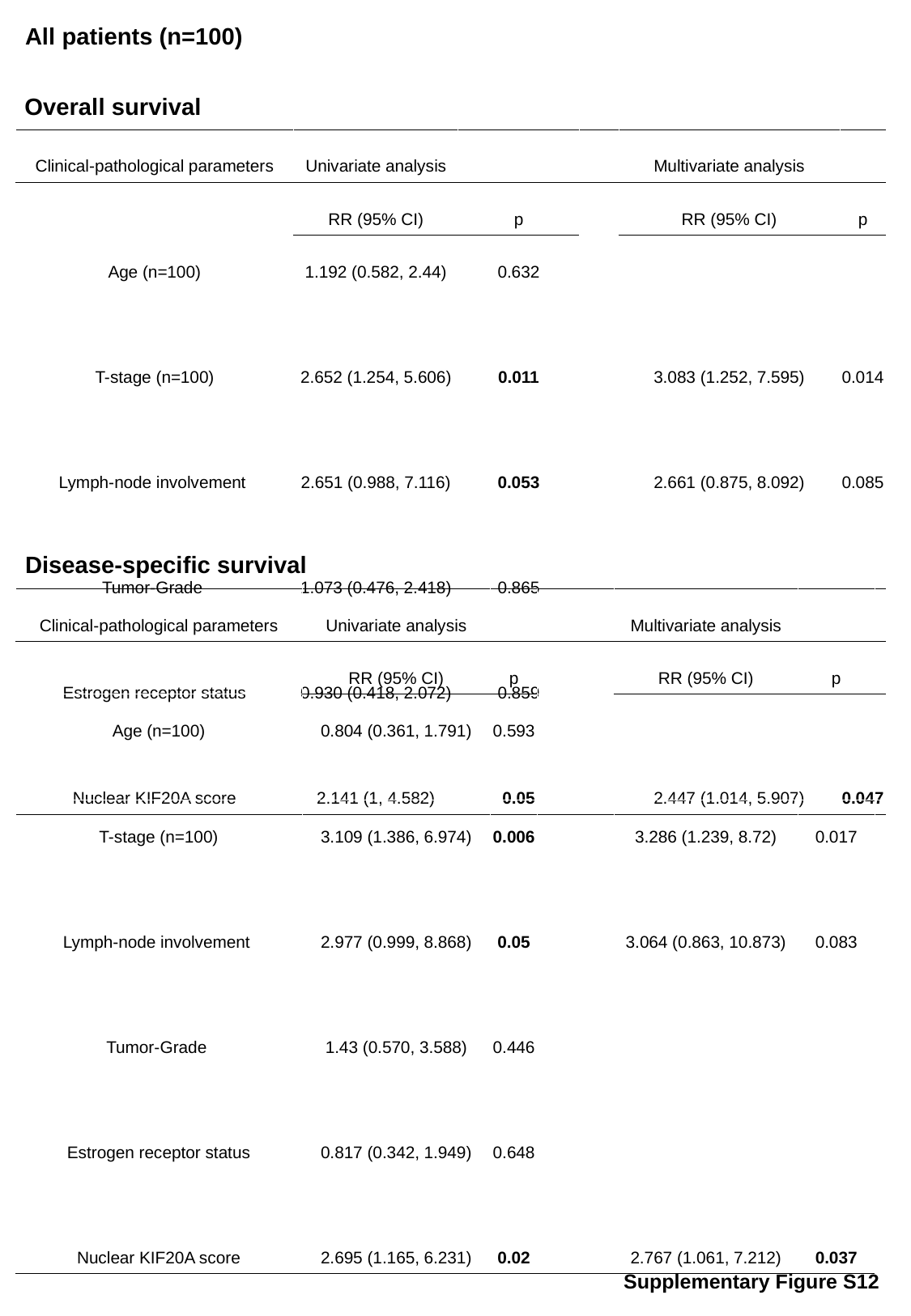

All patients (n=100)
Overall survival
| Clinical-pathological parameters | Univariate analysis | | | Multivariate analysis | |
| --- | --- | --- | --- | --- | --- |
| | RR (95% CI) | p | | RR (95% CI) | p |
| Age (n=100) | 1.192 (0.582, 2.44) | 0.632 | | | |
| | | | | | |
| T-stage (n=100) | 2.652 (1.254, 5.606) | 0.011 | | 3.083 (1.252, 7.595) | 0.014 |
| | | | | | |
| Lymph-node involvement | 2.651 (0.988, 7.116) | 0.053 | | 2.661 (0.875, 8.092) | 0.085 |
| | | | | | |
| Tumor-Grade | 1.073 (0.476, 2.418) | 0.865 | | | |
| | | | | | |
| Estrogen receptor status | 0.930 (0.418, 2.072) | 0.859 | | | |
| | | | | | |
| Nuclear KIF20A score | 2.141 (1, 4.582) | 0.05 | | 2.447 (1.014, 5.907) | 0.047 |
| | | | | | |
| | | | | | |
Disease-specific survival
| Clinical-pathological parameters | Univariate analysis | | | Multivariate analysis | | |
| --- | --- | --- | --- | --- | --- | --- |
| | RR (95% CI) | p | | RR (95% CI) | p | |
| Age (n=100) | 0.804 (0.361, 1.791) | 0.593 | | | | |
| | | | | | | |
| T-stage (n=100) | 3.109 (1.386, 6.974) | 0.006 | | 3.286 (1.239, 8.72) | 0.017 | |
| | | | | | | |
| Lymph-node involvement | 2.977 (0.999, 8.868) | 0.05 | | 3.064 (0.863, 10.873) | 0.083 | |
| | | | | | | |
| Tumor-Grade | 1.43 (0.570, 3.588) | 0.446 | | | | |
| | | | | | | |
| Estrogen receptor status | 0.817 (0.342, 1.949) | 0.648 | | | | |
| | | | | | | |
| Nuclear KIF20A score | 2.695 (1.165, 6.231) | 0.02 | | 2.767 (1.061, 7.212) | 0.037 | |
Supplementary Figure S12
