# Supplementary material for: Paclitaxel targets FOXM1 to regulate KIF20A in mitotic catastrophe and breast cancer paclitaxel resistance
Source: Oncogene. 2015 May 11;35(8):990–1002. doi: 10.1038/onc.2015.152 (PMC4538879; doi:10.1038/onc.2015.152)
Supplement: Supplementary Figure 13 [file onc2015152x16.ppt]

## Slide 1
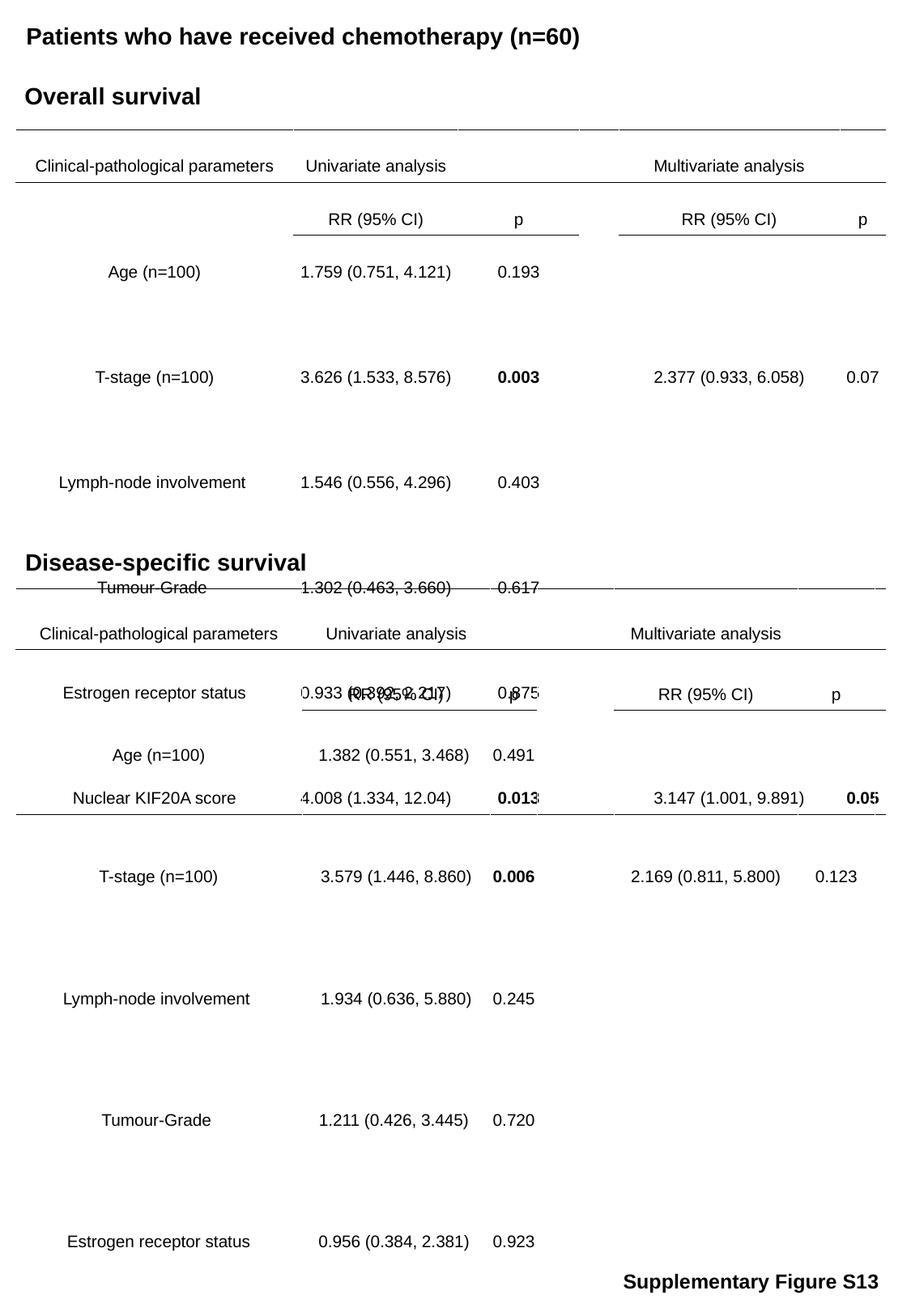

Patients who have received chemotherapy (n=60)
Overall survival
| Clinical-pathological parameters | Univariate analysis | | | Multivariate analysis | |
| --- | --- | --- | --- | --- | --- |
| | RR (95% CI) | p | | RR (95% CI) | p |
| Age (n=100) | 1.759 (0.751, 4.121) | 0.193 | | | |
| | | | | | |
| T-stage (n=100) | 3.626 (1.533, 8.576) | 0.003 | | 2.377 (0.933, 6.058) | 0.07 |
| | | | | | |
| Lymph-node involvement | 1.546 (0.556, 4.296) | 0.403 | | | |
| | | | | | |
| Tumour-Grade | 1.302 (0.463, 3.660) | 0.617 | | | |
| | | | | | |
| Estrogen receptor status | 0.933 (0.392, 2.217) | 0.875 | | | |
| | | | | | |
| Nuclear KIF20A score | 4.008 (1.334, 12.04) | 0.013 | | 3.147 (1.001, 9.891) | 0.05 |
| | | | | | |
| | | | | | |
Disease-specific survival
| Clinical-pathological parameters | Univariate analysis | | | Multivariate analysis | | |
| --- | --- | --- | --- | --- | --- | --- |
| | RR (95% CI) | p | | RR (95% CI) | p | |
| Age (n=100) | 1.382 (0.551, 3.468) | 0.491 | | | | |
| | | | | | | |
| T-stage (n=100) | 3.579 (1.446, 8.860) | 0.006 | | 2.169 (0.811, 5.800) | 0.123 | |
| | | | | | | |
| Lymph-node involvement | 1.934 (0.636, 5.880) | 0.245 | | | | |
| | | | | | | |
| Tumour-Grade | 1.211 (0.426, 3.445) | 0.720 | | | | |
| | | | | | | |
| Estrogen receptor status | 0.956 (0.384, 2.381) | 0.923 | | | | |
| | | | | | | |
| Nuclear KIF20A score | 5.089 (1.467, 17.657) | 0.010 | | 4.108 (1.135, 14.873) | 0.031 | |
Supplementary Figure S13
